# Methods for analysis of complex survey data: an application using the Tanzanian 2015 Demographic and Health Survey and Service Provision Assessment

**DOI:** 10.7189/jogh.09.020902

**Published:** 2019-12

**Authors:** Ashley Sheffel, Emily Wilson, Melinda Munos, Scott Zeger

**Affiliations:** 1 Department of International Health, Johns Hopkins Bloomberg School of Public Health, Baltimore, Maryland, USA; 2 Department of Biostatistics, Johns Hopkins Bloomberg School of Public Health, Baltimore, Maryland, USA

## Abstract

**Background:**

Low-income and middle-income countries (LMICs) seek to better utilize household and health facility survey data for monitoring and evaluation, as well as for health program planning. However, analysis of this complex survey data are complicated. In Tanzania, the National Evaluation Platform project sought to analyze Demographic and Health Survey (DHS) data and Service Provision Assessment (SPA) data as part of an evaluation of the national One Plan for Maternal and Child Health. To support this evaluation, we used this survey data to answer two key methodological questions: 1) what are the benefits and costs of using sampling weights in rate estimation; and 2) what is the best method for calculating standard errors in these two surveys?

**Methods:**

We conducted a simulation study for each methodologic question. The first simulation study assessed the benefits and costs of using sampling weights in rate estimation. This simulation used weighted and unweighted estimates and examined bias, variance, and the mean squared error (MSE). The second simulation study assessed the best method for calculating standard errors comparing cluster bootstrapped variance estimation, design based asymptotic variance with one level (svy1), and design based asymptotic variance with three levels (svy3). We compared coverage probability and confidence interval length.

**Results:**

Our results showed that although weighted estimates were less biased, unweighted estimates were less variable. The weighted estimates had a lower MSE, indicating that the effect of the bias trade-off was greater than the effect of the variance trade-off for most indicators assessed. The best performer for variance estimation was the cluster bootstrap method, followed by the svy3 method. The svy1 method was the worst performer for most indicators assessed.

**Conclusions:**

As complex survey data become more widely used for policymaking in LMICs, there is a need for guidance on the best methods for analyzing this data. The standard of practice has been a design-based analysis using survey weights and the single-level svy method for calculating standard errors. This study puts forth an alternative approach to analysis. In addition, this study offers practical guidance on determining the best method for analysis of complex survey data.

Low-income and middle-income countries (LMICs) increasingly use complex survey data from household and health facility surveys to monitor and evaluate progress toward health targets as well as for program planning. However, there are several barriers to using this data, including low capacity and insufficient skills to analyze and interpret the data. LMICs also lack guidance on the best methods for analyzing these data, because analysis of complex survey data are not straightforward [1].

Sample surveys take a random sample of the population in order to make inferences about the entire population from which the sample was taken. The most information-efficient method is to take a simple random sample of the population. However, this method is not always logistically possible. Consequently, more complex sampling designs are often employed, utilizing clustering and stratification, generally leading to unequal probability of selection. In the analysis phase, the design of the survey is taken into account to ensure the results reflect the population.

The standard practice for analyzing survey data are to use statistical software packages to address differences between the survey design and simple random sampling [2]. These packages require the user to specify the primary, secondary, or tertiary sampling units, as applicable (PSUs, SSUs, and TSUs), sampling weights, and strata as well as the desired variance estimator. The sampling weights are applied to point estimates while stratification and/or clustering are taken into account when calculating standard errors.

However, there is considerable debate among survey sampling experts regarding the best analytical methods for complex survey data [3-6]. Ideally, both bias and variance would be minimized in the analysis of complex survey data, but there is a trade-off between bias and variance when choosing to weight data. Weighting data is inefficient, as it discards some data and thus increases the variance of estimates. However, researchers are often willing to accept inefficiency to obtain unbiased estimates. There is also a lack of consensus about the best approach for variance estimation when working with complex survey data. Variance provides information about the precision of the point estimate and is important for calculation of confidence intervals and hypothesis tests. Accordingly, the variance estimation method should ideally account for both the sampling design and the correlation created by the nested nature of the data in order to produce valid estimates of variance.

This debate generated methodological questions in the context of the National Evaluation Platform (NEP) in Tanzania, which conducted an evaluation of the National One Plan for Maternal and Child Health (“One Plan”). The NEP is a project aimed at building national capacity for generating evidence-based answers to program and policy questions using extant data. The evaluation planned to assess the One Plan’ s approach to reducing maternal mortality, focusing on antenatal care (ANC) and including assessments of ANC intervention coverage and service quality. The main data source for measuring intervention coverage was the Tanzania DHS (TDHS), implemented in 2004, 2010, and 2015. The main data source for measuring service quality was the Tanzania SPA (TSPA), implemented in 2006 and 2015. In order to analyze these complex survey data, the Tanzanian NEP team required methodological guidance. This type of guidance is important for LMIC governments in order to overcome barriers to data analysis and data use for decision-making.

This study used the 2015 TDHS data and the 2015 TSPA data on ANC to provide methodological guidance for the NEP Tanzania team and for others using similar data sources. We answered two key questions: 1) what are the benefits and costs of using sampling weights in rate estimation; and 2) what is the best method for calculating standard errors in these two surveys? We investigated how weights affect both the bias and the variance of an estimate; we also assessed whether weighted or unweighted estimates are better able to minimize the combination of bias and variance. In addition, we evaluated multiple methods for variance estimation to determine which is the best estimator of the standard error.

## METHODS

### Data

We used the 2015 TDHS and the 2015 TSPA, both publicly available data sets, for this analysis. Access to the 2015 TDHS and the 2015 TSPA was granted through the DHS program.

#### Demographic and Health Survey (DHS)

Data were collected using four types of survey instruments: a household questionnaire, a women’s questionnaire, a men’s questionnaire, and a biomarker questionnaire. The TDHS final report contains comprehensive information on the survey methodology and the questionnaires [7].

The TDHS employed a multi-stage cluster sampling approach stratified by region and urban/rural designation. In the first stage, the sampling frame for each stratum was comprised of the listing of census enumeration areas (EAs) defined by the 2012 Tanzania Population and Housing Census. EAs were selected using systematic random sampling with probability proportional to size. In the second stage, the sampling frame was comprised of a complete listing of households from the selected clusters. A fixed number of households were then sampled using systematic random sampling in each cluster. All household members in the selected households who met the eligibility criteria for the men’s and women’s questionnaires were included in the survey.

TDHS survey weights were calculated by the DHS program and were included in the final data set. The household weight was calculated based on the household selection probability, adjusted for non-response. The individual weight was calculated based on the household weight multiplied by the inverse of the individual response rate. The Guide to DHS Statistics contains more detailed information on sampling and the calculation of weights for DHS surveys [8].

#### Service Provision Assessment (SPA)

Data were collected using four types of survey instruments: a facility inventory questionnaire, health worker interviews, observation of ANC consultations, and exit interviews with ANC clients. The TSPA final report contains comprehensive information on the survey methodology and questionnaires [9].

The TSPA sampling frame was comprised from a master facility list (MFL) compiled by the Ministry of Health, Community Development, Gender, Elderly, and Children (MOHCDGEC) on mainland Tanzania and the Ministry of Health in Zanzibar. The NEP analysis was restricted to mainland Tanzania because Zanzibar functions under a separate government; therefore, this analysis was also restricted to mainland Tanzania. Strata were established by crossing region (25 mainland regions) and facility type (hospital, health center, dispensary, and clinic). These strata were then used to select health facilities by stratified systematic probability sampling. In addition, hospitals were oversampled to include all hospitals in the country.

The health worker sampling frame was comprised of the list of providers who were present on the day of assessment and who provided services assessed by the SPA survey. In facilities with fewer than eight providers, all providers were interviewed. In larger facilities, all providers whose consultations were observed and providers who gave information for any section of the facility inventory questionnaire were interviewed. Subsequently, a random selection of the remaining providers in the facility was interviewed to obtain a total of eight providers.

The client sampling frame was comprised of the expected number of ANC clients present on the day of the survey as reported by the health facility. Clients were randomly selected for observation during their visit based on the expected number of ANC clients on the day of the visit. In facilities where the number of expected ANC clients could not be predetermined, an opportunistic sample was taken. Observation of client-provider interactions was completed for a maximum of five clients per service provider, with a maximum of 15 observations in any given facility. Exit interviews were conducted with all clients whose visits were observed. For client-provider observations, the NEP analysis, and therefore this analysis, was restricted to first visit ANC observations as it was difficult to determine the exact package of services that should be delivered at subsequent ANC visits.

SPA survey weights were calculated by the DHS program and included in the final data set. The health facility weight was calculated based on the health facility selection probability, adjusted for non-response at the sampling stratum level. The provider weight was the product of the facility weight and the inverse selection probability of providers within each of the sampling strata, adjusted for provider non-response. The provider weight takes into account differentials caused by over-sampling or under-sampling of providers with a particular professional qualification in each stratum. Client weights were calculated by taking the facility weight multiplied by the inverse selection probability of clients within each of the sampling strata, adjusted for client non-response.

### Selection of indicators

We selected ten indicators from the TSPA data set related to ANC. These indicators were selected to capture data at facility and client levels, and to examine varying point estimates. In addition, we selected four indicators from the TDHS data set related to ANC. Table S1 in **Online Supplementary Document** shows the selected indicators, weighted national means, as well as the minimum and maximum regional weighted means for the indicators.

### Analysis

**Figure 1:**
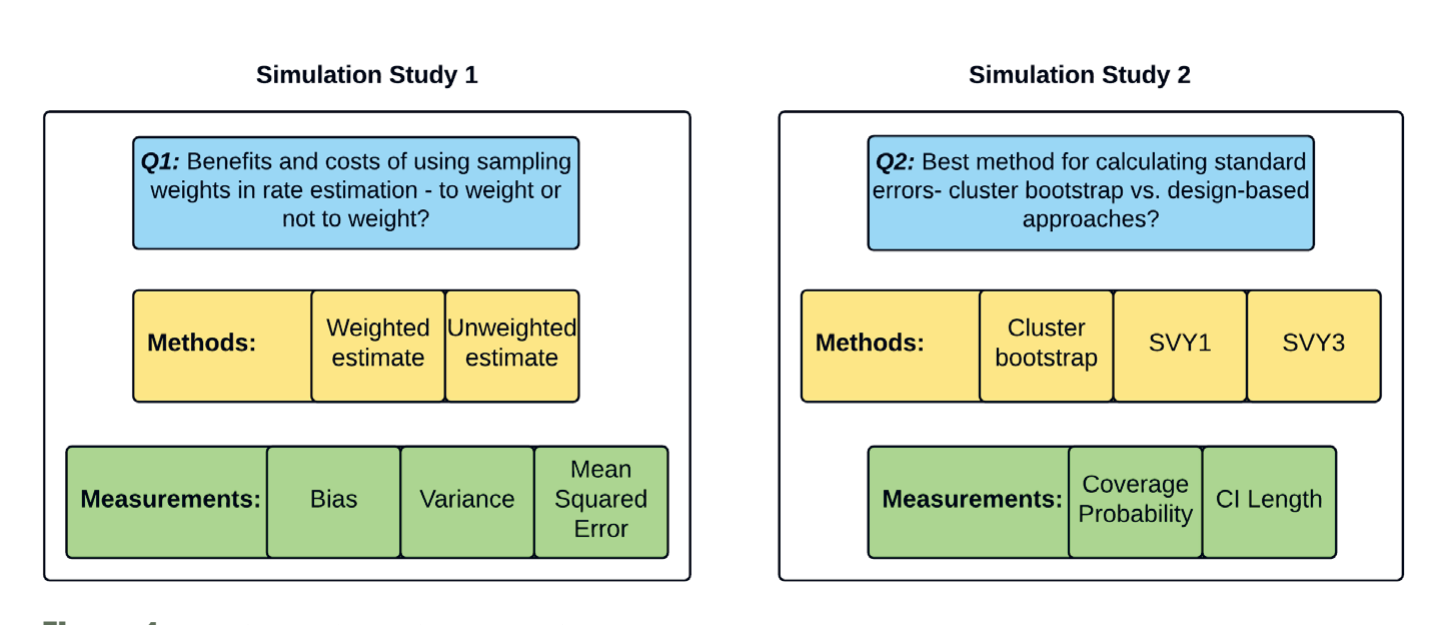
Study analytical approach.

The analytical approach for this study is depicted in **Figure 1****.**

#### Simulation study

We used individuals from the 2015 TDHS women’s data set as the population from which to draw a new sample. We randomly sampled women with replacement (ie, individuals could be selected more than once) from the TDHS 2015 survey, but stratified the resampling by region so that each new sample had the same number of individuals per region as in the original survey. We generated 500 independent survey samples. The number 500 was chosen so that the estimates of coverage probability for 95% confidence intervals had a standard error less than 0.01. We treated the original 2015 TDHS survey as the “truth” about the population of women in Tanzania and compared it to the 500 simulated sample surveys.

We took the same simulation approach at the facility level. We used health facilities from the 2015 TSPA as the population of health facilities from which we drew a new sample. We randomly sampled facilities with replacement (ie, health facilities could be selected more than once) from the TSPA 2015 survey and selected the same number of facilities per region as in the original survey. For client-level analyses, we merged the resampled facilities with the client data for those facilities to create a client-level data set. We repeated this process 500 times to create 500 different simulated sample surveys. We treated the original 2015 TSPA survey as the “truth” about the population of health facilities in Tanzania and compared it to the 500 simulated sample surveys.

#### Weighting analysis for simulation study

This analysis compared two methods for calculating point estimates: weighted means and unweighted means. We used the weights provided in the TDHS and TSPA data sets to calculate weighted means. For each of the 500 simulated samples, we calculated a weighted and an unweighted mean by region and at the national level for the indicators of interest. We also calculated the weighted “true” means of these indicators from the original surveys by region and at the national level. We compared the bias, variance, and MSE of the weighted and unweighted means at regional and national levels. Bias was calculated as the average over the 500 replicates of the (un)weighted simulation mean minus the “true” weighted mean. Variance was calculated as the mean of the squared differences between the estimates and their means. The MSE was calculated as bias squared plus variance.

#### Variance analysis for simulation study

This analysis compared three methods for calculating standard errors: 1) clustered bootstrap variance, 2) design-based asymptotic variances from the R package “survey” with one level (svy1), and 3) design-based asymptotic variances from the R package “survey” with three levels (svy3). The R “survey” package is a design-based analytical package for survey data similar to the “svy” package in STATA. All analyses used the weights provided in the TSPA data set. This analysis was conducted only for the TSPA indicators. The TDHS did not have the required variables for the svy3 analysis and the bootstrap variance estimation was not feasible due to the amount of time it would take to run the analysis.

**Method 1- bootstrap variance:** The bootstrap variance method was implemented by resampling facilities with replacement and then merging the sampled facilities with client data to get sampled clients. A simple weighted average was calculated by region for the indicators of interest. This process was repeated 100 times. The estimates were averaged to produce point estimates. The square root of the sample variance of the 100 estimates was taken as the standard errors by regions. In addition, bootstrapped confidence intervals were obtained from the 0.025 and 0.975 percentiles across the 100 replicates. Because we resampled facilities, not individuals, this bootstrapping approach accounts for the clustering of clients within providers and providers within facilities.

**Method 2- svy1:** The single-level svy method utilized the “survey” package to specify the survey design. The primary sampling unit (PSU) was the facility, the sampling weight was the facility weight or client weight, and the strata was the combination of region and facility type. For facility-level indicators, the facility weight was used, and for client-level indicators the client weight was used. This design did not account for clustering of clients within providers and providers within facilities. This approach is the DHS-recommended method [10].

**Method 3- svy3:** The multi-level svy method also utilized the “survey” package to specify the survey design. The PSU was the facility, the SSU was the provider, and the TSU was the client. The strata were the combination of region and facility type. The finite population correction factor (fpc) was specified at facility, provider, and client levels. The svy3 method was only applied to client-level indicators as facility-level indicators did not have multiple sampling stages. This design accounted for the clustering of clients within providers and providers within facilities, as well as for the finite population correction factor. The fpc is particularly relevant in the analysis of SPA data as the sample size is greater than five percent of the population size.

We compared the three methods for obtaining confidence intervals in terms of coverage probabilities and average confidence interval lengths by region and at the national level. The actual coverage probability was estimated by the proportion of the 500 simulation surveys in which the nominal 95% confidence interval contained the true value. The simulation sample size was n = 500, producing a standard error of 0.01 for a 95% coverage probability. Therefore, we determined a coverage probability of 0.93-0.97 (±2 standard errors) to be good coverage, a coverage probability of 0.91-0.93 and >0.97 to be fair coverage, a coverage probability of less than 0.91 to be poor coverage. The average confidence interval length was calculated by the average of the widths of the confidence intervals across the 500 simulations.

All statistical analyses were carried out using R version 3.3.3 [11].

### Alternative methods

Prior to establishing bootstrapping as the most appropriate variance estimation method to compare with the design-based methods, we attempted other variance estimation methods. First, we used a mixed-effects logistic regression implemented with the R package “glmer.” This option was attractive as it allowed us to utilize individual-level data from the simulated surveys and to specify random effects for facilities and providers in each region. However, in some cases the model failed to converge, producing unreasonable estimates of standard errors. We resolved this problem by specifying two additional options in the glmer model. We set the glmer starting value to the region’s “true” weighted rate and we used the bobyqa optimizer rather than the package default. Although these changes resolved the convergence issue, the model took so long to run that it was deemed impractical to pursue this method further.

## RESULTS

### Survey Characteristics

The 2015 TDHS data set contained data from 10 808 households in mainland Tanzania representing 11 127 women aged 15-49, of whom 6078 had a live birth in the five years preceding the survey (**Table 1**).

**Table 1:** Sample size information, TDHS 2015, mainland Tanzania

Region	Total number of households	Total number of women ages 15-49	Total number of women ages 15-49 with a live birth in the 5 years preceding the survey
Arusha	418	420	216
Dar es salaam	700	797	341
Dodoma	420	343	197
Geita	431	535	303
Iringa	415	340	163
Kagera	439	416	239
Katavi	401	466	315
Kigoma	432	491	279
Kilimanjaro	449	370	130
Lindi	423	380	185
Manyara	426	434	251
Mara	438	531	321
Mbeya	410	374	192
Morogoro	398	345	194
Mtwara	410	348	161
Mwanza	406	496	254
Njombe	406	359	185
Pwani	385	333	184
Rukwa	436	425	272
Ruvuma	434	383	214
Shinyanga	421	516	311
Simiyu	420	587	372
Singida	438	413	245
Tabora	426	560	338
Tanga	426	465	216
National	10,808	11,127	6,078

TDHS – Tanzanzia Demographic and Health Survey

The 2015 TSPA data set contained data from 1078 health facilities in mainland Tanzania, of which 949 provided ANC services. From the facilities that provided ANC services, 741 facilities had at least one ANC client observation (**Table 2**). Across all facilities, 994 ANC providers had observed consultations. On average, each facility had 4.9 ANC client observations. The number of ANC consultations at a health facility ranged from 1 to 15. Across all mainland Tanzania health facilities there were 3641 ANC consultations with 1607 of these consultations being first ANC visits for a pregnant woman.

**Table 2:** Sample Size Information, TSPA 2015, Mainland Tanzania

Region	Total number of facilities	Total number of facilities offering ANC	Total number of facilities offering ANC with at least 1 ANC client observation	Total number of ANC providers with observed consultations	Total number of ANC client observations	Total number of first visit ANC client observations
Arusha	45	39	32	42	147	63
Dar es salaam	87	49	33	42	159	70
Dodoma	48	45	38	60	218	42
Geita	35	30	27	33	92	70
Iringa	39	36	32	37	125	61
Kagera	44	41	37	61	236	101
Katavi	35	32	24	34	132	88
Kigoma	41	40	30	43	181	41
Kilimanjaro	52	43	27	38	140	111
Lindi	37	37	35	48	190	70
Manyara	33	32	24	37	127	107
Mara	41	38	38	51	217	46
Mbeya	53	51	22	38	116	32
Morogoro	49	46	25	30	88	76
Mtwara	37	34	27	36	145	76
Mwanza	46	36	34	40	147	51
Njombe	39	37	42	44	164	47
Pwani	41	34	27	30	107	45
Rukwa	37	34	29	38	124	98
Ruvuma	42	41	33	49	196	33
Shinyanga	35	28	28	34	100	27
Simiyu	34	31	20	22	69	54
Singida	37	32	25	32	129	59
Tabora	44	42	26	34	133	75
Tanga	47	41	26	41	159	64
National	1,078	949	741	994	3,641	1,607

ANC – antenatal care, TSPA – Tanzania Service Provision Assessment

### Q1: The benefits and costs of using sampling weights in rate estimation — to weight or not to weight?

**Table 3** shows comparisons of bias, variance, and MSE for weighted vs unweighted estimates for the fourteen indicators at national level. **Tables 4-5** show the comparison of bias, variance, and MSE for weighted vs unweighted estimates for one indicator from the TDHS (Percentage of women attended four or more times during pregnancy by any provider (ANC4+)) and one indicator from the TSPA (Client had no problem with the amount of time waited) with regional disaggregation. In addition, Tables S2-S13 in **Online Supplementary Document** contain regional disaggregation for the remaining twelve indicators.

**Table 3:** Comparison of bias, variance, and MSE for weighted vs unweighted estimates for 14 indicators, nationally, TSPA 2015 and TDHS 2015

				BIAS		VARIANCE					MEAN SQUARED ERROR (MSE)			
**Column 1**	**2**	**3**	**4**	**5**	**6**	**7**	**8**	**9**	**10**	**11**	**12**	**13**	**14**	**15**
**Indicator name**	**Weighted TRUTH ( × 10^2^)**	**Average unweighted simulation estimate ( × 10^2^)**	**Average weighted simulation estimate ( × 10^2^)**	**Mean difference in bias ( × 10^2^)**	**95% CI mean difference in bias**	**Variance of unweighted estimate ( × 10^3^)**	**Variance of weighted estimate ( × 10^3^)**	**Log of the ratio of the variances (unweighted/weighted)**	**95% CI of log of the ratio of the variances**	**Ratio of the variances (unweighted/weighted)**	**MSE of unweighted estimates ( × 10^3^)**	**MSE of weighted estimates ( × 10^3^)**	**Log of the ratio of the MSEs (unweighted/weighted)**	**95% CI of log of the ratio of the MSEs**
Client had no problem with the amount of time waited	70.0	66.3	69.8	-3.6*	-5.6, -1.4	0.1	0.2	-0.701*	-0.733, -0.478	0.496	1.5	0.2	1.278*	1.595, 1.922
Client had no problem with privacy from having others hear	96.8	96.8	96.8	0	-0.6, 0.7	0.0	0.0	-0.536*	-0.726, -0.518	0.585	0.0	0.0	-0.595*	-0.69, -0.553
Client had no problem with the cleanliness of the facility	85.3	85.7	85.4	0.3	-1.2, 1.9	0.1	0.1	-0.453*	-0.564, -0.314	0.636	0.1	0.1	-0.326*	-0.532, -0.321
Provider weighed the client	80.8	86.0	80.8	5.2*	1.8, 8.7	0.2	0.7	-1.172*	-1.36, -1.134	0.310	2.9	0.7	0.811*	0.734, 0.951
Provider asked about or performed syphilis test	13.7	17.4	13.6	3.8*	1.4, 6.2	0.2	0.3	-0.129	-0.192, 0.042	0.879	1.6	0.3	1.268*	1.255, 1.536
Provider provided or prescribed tetanus toxoid vaccine	53.5	58.1	53.6	4.5*	1.1, 8	0.3	0.6	-0.922*	-0.937, -0.671	0.398	2.4	0.6	0.746*	0.598, 0.772
Hemoglobin test available at the facility	29.5	52.0	29.4	22.6*	19.7, 25.5	0.3	0.4	-0.269*	-0.493, -0.224	0.764	51.1	0.4	4.258*	4.256, 4.515
TT vaccine available at the facility	86.4	90.3	86.4	3.9*	1.8, 5.9	0.1	0.3	-1.109*	-1.27, -1.029	0.330	1.6	0.3	1.135*	1.106, 1.236
IPT drug available at the facility	63.2	67.1	63.3	3.8*	0.8, 7.1	0.2	0.5	-0.854*	-1.043, -0.799	0.426	1.8	0.5	0.678*	0.578, 0.743
ITNs or ITN vouchers available at facility	9.8	17.7	9.8	7.9*	5.8, 9.9	0.2	0.2	-0.016	-0.258, 0.028	0.984	6.4	0.2	2.944*	2.824, 3.023
ANC1 coverage	98.0	98.0	98.0	0	-0.2, 0.3	0.0	0.0	-0.384*	-0.415, -0.222	0.681	0.0	0.0	-0.292*	-0.294, -0.124
ANC4+ coverage	50.9	49.3	50.9	-1.6*	-2.5, -0.5	0.1	0.1	-0.328*	-0.42, -0.248	0.720	0.3	0.1	0.678*	0.627, 0.772
IPTp coverage	35.8	35.3	35.8	-0.5	-1.4, 0.3	0.1	0.1	-0.345*	-0.435, -0.27	0.708	0.1	0.1	-0.159*	-0.276, -0.117
Iron supplementation coverage	21.8	20.9	21.8	-0.9*	-1.7, -0.2	0.0	0.1	-0.315*	-0.416, -0.233	0.730	0.1	0.1	0.392*	0.357, 0.56

MSE – mean squared error, CI – confidence interval, ANC – antenatal care, IPTp – intermittent preventive treatment of malaria during pregnancy

*Indicates *P*-value <0.05.

**Table 4:** Comparison of bias, variance, and MSE for weighted vs unweighted estimates for the household survey indicator “ANC4+ Coverage,” by region

				BIAS		VARIANCE					MEAN SQUARED ERROR (MSE)			
**Column 1**	**2**	**3**	**4**	**5**	**6**	**7**	**8**	**9**	**10**	**11**	**12**	**13**	**14**	**15**
**Region**	**Weighted TRUTH ( × 10^2^)**	**Average unweighted simulation estimate ( × 10^2^)**	**Average weighted simulation estimate ( × 10^2^)**	**Mean difference in bias ( × 10^2^)**	**95% CI mean difference in bias**	**Variance of unweighted estimate ( × 10^3^)**	**Variance of weighted estimate ( × 10^3^)**	**Log of the ratio of the variances (unweighted/weighted)**	**95% CI of log of the ratio of the variances**	**Ratio of the variances (unweighted/weighted)**	**MSE of unweighted estimates ( × 10^3^)**	**MSE of weighted estimates ( × 10^3^)**	**Log of the ratio of the MSEs (unweighted/weighted)**	**95% CI of log of the ratio of the MSEs**
Arusha	50.1	50.4	50.2	0.2	-1.2, 1.7	2.0	1.9	0.051*	(0.012, 0.08)	1.053	2.0	1.9	0.047*	0.023, 0.062
Dar es Salaam	75.0	73.7	74.9	-1.2*	-2.6, -0.1	0.4	0.5	-0.159*	(-0.238, -0.133)	0.853	0.6	0.5	-0.001	-0.02, 0.148
Dodoma	58.4	59.5	58.5	1.1	-0.9, 3.1	2.4	2.4	0.012	(-0.034, 0.044)	1.012	2.5	2.4	0.025*	0.018, 0.098
Geita	37.9	37.4	38.0	-0.6	-2.5, 0.9	1.3	1.3	-0.001	(-0.073, 0.01)	0.999	1.3	1.3	-0.046*	-0.117, -0.007
Iringa	56.8	57.4	56.6	0.8	-1.2, 2.9	2.5	2.3	0.092*	(0.062, 0.14)	1.097	2.6	2.3	0.111*	0.084, 0.133
Kagera	46.9	48.7	47.0	1.7	-0.2, 3.9	2.0	2.2	-0.13*	(-0.156, -0.075)	0.878	2.3	2.2	-0.076*	-0.233, -0.076
Katavi	32.9	34.7	33.2	1.5	-0.3, 3.4	1.3	1.1	0.109*	(0.045, 0.143)	1.115	1.6	1.1	0.241	-0.101, 0.268
Kigoma	21.1	28.1	21.4	6.7*	3.7, 10.2	2.1	1.8	0.116*	(0.05, 0.199)	1.123	6.9	1.9	0.84*	0.766, 0.939
Kilimanjaro	54.8	57.1	55.0	2	-0.3, 4.7	1.8	2.1	-0.191*	(-0.244, -0.142)	0.826	2.3	2.1	-0.067	-0.1, 0.024
Lindi	54.3	54.0	54.3	-0.4	-2.9, 2.2	1.5	1.7	-0.192*	(-0.194, -0.096)	0.825	1.5	1.7	-0.124*	-0.162, -0.12
Manyara	55.1	57.6	55.3	2.3	-1.5, 7.2	1.4	2.4	-0.444*	(-0.566, -0.418)	0.642	2.1	2.4	-0.262*	-0.26, -0.157
Mara	48.9	47.4	48.5	-1.1	-3, 1	1.2	1.4	-0.153*	(-0.223, -0.135)	0.858	1.4	1.5	-0.105*	-0.233, -0.105
Mbeya	46.0	47.3	46.6	0.7	-4.1, 4.6	2.6	3.1	-0.302*	(-0.269, -0.116)	0.739	2.8	3.2	-0.147*	-0.163, -0.04
Morogoro	71.7	71.1	71.7	-0.6	-2.5, 1.1	0.9	0.9	0.051	(-0.028, 0.075)	1.052	0.9	0.9	0.036	-0.086, 0.045
Mtwara	51.0	52.6	51.5	1.1*	0, 2.3	1.8	1.8	0.024	(-0.017, 0.03)	1.024	2.0	1.8	0.091*	0.086, 0.146
Mwanza	42.1	39.1	41.7	-2.5	-5.7, 1.2	2.4	3.1	-0.306*	(-0.315, -0.212)	0.736	3.3	3.2	-0.109	-0.124, 0.055
Njombe	49.9	49.6	50.2	-0.7	-1.9, 0.3	1.9	1.8	0.077*	(0.038, 0.083)	1.081	2.0	1.8	0.063*	0.023, 0.065
Pwani	71.5	71.5	71.6	-0.1	-1.3, 1.2	0.9	0.8	0.203*	(0.168, 0.233)	1.225	0.9	0.8	0.172*	0.134, 0.203
Rukwa	47.1	43.6	47.0	-3.3*	-5.1, -1.7	2.1	2.3	-0.12*	(-0.12, -0.052)	0.887	3.3	2.3	0.157*	0.063, 0.185
Ruvuma	43.6	43.9	44.0	0	-1.5, 1.9	1.5	1.4	0.012	(-0.014, 0.06)	1.012	1.5	1.4	0.015*	0.001, 0.039
Shinyanga	50.1	48.8	50.1	-1.2	-3.1, 0.9	2.6	3.0	-0.176*	(-0.184, -0.119)	0.839	2.7	3.0	-0.107*	-0.131, -0.069
Simiyu	39.1	39.1	39.0	0.2	-3.1, 3.7	1.1	1.4	-0.179*	(-0.265, -0.106)	0.836	1.1	1.4	-0.18*	-0.291, -0.167
Singida	53.6	53.2	53.6	-0.4	-2.5, 1.5	0.8	0.8	-0.119*	(-0.138, -0.006)	0.888	0.8	0.8	-0.044	-0.183, 0.029
Tabora	39.1	39.5	39.2	0.3	-1.1, 2.2	1.9	1.8	0.05*	(0.031, 0.097)	1.051	1.9	1.8	0.066*	0.045, 0.108
Tanga	62.7	61.4	62.6	-1.1	-3.5, 0.9	1.6	1.6	-0.013	(-0.06, 0.037)	0.987	1.8	1.6	0.027*	0.025, 0.145
National	50.9	49.3	50.9	-1.6*	-2.5, -0.5	0.1	0.1	-0.328*	(-0.42, -0.248)	0.720	0.3	0.1	0.678*	0.627, 0.772

MSE – mean squared error, CI – confidence interval

*Indicates *P*-value <0.05.

**Table 5:** Comparison of bias, variance, and MSE for weighted vs unweighted estimates for the facility survey indicator “client had no problem with the amount of time waited,” by region

				BIAS		VARIANCE						MEAN SQUARED ERROR (MSE)				
**Column 1**	**2**	**3**	**4**	**5**	**6**	**7**	**8**	**9**	**10**	**11**		**12**	**13**	**14**		**15**
**Region**	**Weighted TRUTH ( × 10^2^)**	**Average unweighted simulation estimate ( × 10^2^)**	**Average weighted simulation estimate ( × 10^2^)**	**Mean difference in bias ( × 10^2^)**	**95% CI mean difference in bias**	**Variance of unweighted estimate ( × 10^3^)**	**Variance of weighted estimate ( × 10^3^)**	**Log of the ratio of the variances (unweighted/weighted)**	**95% CI of log of the ratio of the variances**		**Ratio of the variances (unweighted/weighted)**	**MSE of unweighted estimates ( × 10^3^)**	**MSE of weighted estimates ( × 10^3^)**	**Log of the ratio of the MSEs (unweighted/weighted)**	**95% CI of log of the ratio of the MSEs**	
Arusha	65.8	64.3	66.2	-1.9	-8.8, 6.8	3.0	4.1	-0.325*	(-0.44, -0.215)		0.723	3.2	4.1	-0.282*	-0.403, -0.115	
Dar es Salaam	75.3	63.1	74.7	-11.6*	-18.4, -3.4	1.8	1.9	-0.086	(-0.172, 0.12)		0.917	16.7	1.9	1.55*	1.909, 2.161	
Dodoma	63.6	60.6	63.2	-2.5	-8.4, 3.6	2.8	3.1	-0.134*	(-0.2, -0.028)		0.874	3.6	3.1	0.057*	0.182, 0.342	
Geita	53.1	45.5	52.3	-6.7	-14, 1.3	4.3	6.7	-0.498*	(-0.518, -0.356)		0.608	10.0	6.8	0.056*	0.107, 0.408	
Iringa	82.5	78.5	82.1	-3.6	-11.3, 5.4	2.3	2.8	-0.165*	(-0.349, -0.07)		0.848	3.9	2.8	0.092*	0.288, 0.748	
Kagera	59.9	62.2	60.0	2.2	-8.4, 10.9	1.4	4.6	-1.152*	(-1.288, -1.048)		0.316	1.9	4.6	-1.027*	-0.949, -0.536	
Katavi	69.8	65.6	69.7	-4.1	-9, 1	3.2	2.7	0.228*	(0.083, 0.282)		1.256	5.0	2.7	0.429*	0.605, 1.365	
Kigoma	82.7	77.1	82.7	-5.5	-11.4, 0.5	2.6	1.3	0.648*	(0.589, 0.803)		1.912	5.7	1.3	1.158*	1.433, 1.832	
Kilimanjaro	71.9	70.5	71.7	-1.2	-4.1, 0	1.5	1.6	-0.032*	(-0.102, -0.004)		0.968	1.7	1.6	0.01*	0.029, 0.182	
Lindi	57.0	57.6	56.3	1.2	-4.3, 7.6	3.5	5.4	-0.475*	(-0.495, -0.361)		0.622	3.6	5.5	-0.455*	-0.491, -0.35	
Manyara	77.8	63.9	78.0	-14*	-23.7, -3.5	4.4	5.2	-0.2*	(-0.296, -0.047)		0.819	23.6	5.2	0.99*	1.463, 1.965	
Mara	69.3	65.7	68.8	-3.1	-10.1, 3.8	2.1	2.8	-0.37*	(-0.378, -0.189)		0.690	3.5	2.9	0.033*	0.165, 0.541	
Mbeya	77.0	75.2	77.4	-2.2	-12, 8.1	2.4	4.2	-0.47*	(-0.682, -0.403)		0.625	2.8	4.2	-0.485*	-0.667, -0.351	
Morogoro	70.1	69.3	70.2	-0.9	-9.2, 10.5	4.1	7.6	-0.75*	(-0.715, -0.532)		0.472	4.2	7.6	-0.619*	-0.679, -0.446	
Mtwara	65.1	60.8	65.4	-4.6	-11.6, 1.9	3.4	6.1	-0.603*	(-0.642, -0.502)		0.547	5.3	6.1	-0.353*	-0.339, -0.074	
Mwanza	86.4	78.8	86.5	-7.7*	-14, -2.4	1.2	0.9	0.35*	(0.215, 0.481)		1.419	7.1	0.9	1.539*	1.467, 2.015	
Njombe	91.6	86.3	91.5	-5.2*	-10.1, -1	2.0	1.0	0.669*	(0.589, 0.745)		1.952	4.8	1.0	1.281*	1.674, 1.932	
Pwani	51.5	45.1	51.1	-6.1*	-11.8, -0.1	1.8	3.3	-0.577*	(-0.736, -0.537)		0.561	6.0	3.4	0.106*	0.447, 1.234	
Rukwa	60.1	54.5	60.4	-5.9	-14.9, 3.2	3.3	5.7	-0.526*	(-0.667, -0.46)		0.591	6.4	5.7	-0.16*	0.089, 0.474	
Ruvuma	84.2	80.2	84.0	-3.8	-8.9, 1.4	1.4	1.6	-0.073*	(-0.225, -0.002)		0.930	3.0	1.6	0.34*	0.653, 1.398	
Shinyanga	45.2	39.1	44.9	-5.8	-18.3, 6.9	5.3	7.3	-0.293*	(-0.457, -0.209)		0.746	9.0	7.3	-0.051	-0.548, 0.187	
Simiyu	75.0	75.3	74.9	0.3	-8.4, 9.8	2.0	6.3	-1.126*	(-1.219, -1.046)		0.324	2.0	6.3	-1.113*	-1.244, -1.006	
Singida	65.6	70.6	65.7	4.9	-2.1, 13.7	2.6	4.6	-0.573*	(-0.651, -0.451)		0.564	5.1	4.6	-0.197	-0.229, 0.088	
Tabora	73.8	69.1	73.3	-4.1	-10.8, 1.8	2.5	2.6	-0.039	(-0.136, 0.068)		0.962	4.7	2.7	0.333*	0.384, 0.72	
Tanga	49.6	50.7	51.4	-0.7	-13.3, 12.1	4.8	12.6	-0.916*	(-1.049, -0.864)		0.400	4.9	13.0	-0.947*	-0.969, -0.863	
National	70.0	66.3	69.8	-3.6*	-5.6, -1.4	0.1	0.2	-0.701*	(-0.733, -0.478)		0.496	1.5	0.2	1.278*	1.595, 1.922	

MSE – mean squared error, CI – confidence interval

*Indicates *P* value <0.05.

Column 1 contains either the indicator name (**Table 3**) or the region name (**Table 4** and **Table 5****,** and Tables S2-S13 in **Online Supplementary Document*****)***. Columns 2-4 contain information on the point estimates obtained from the original survey data set (column 2), the average unweighted simulation point estimate (column 3), and the average weighted simulation point estimate (column 4).

Columns 5-6 contain the estimated difference in bias between the weighted and unweighted estimates and the 95% confidence interval for each indicator (**Table 3**) or region (**Table 4** and **Table 5**, and Tables S2-S13 in **Online Supplementary Document**). As we defined the observed weighted mean as the true value, the weighted estimate is an unbiased estimator of the population truth. Column 5 values are the degree of over- or under-estimation of the truth obtained when implementing an unweighted analysis. For example, we see in **Table 3** that the unweighted estimate for hemoglobin overestimates the availability of hemoglobin testing by 22.6% whereas the unweighted estimate for the proportion of ANC clients who had no problem with the amount of time waited is an underestimation of 3.6%.

Columns 7-11 contain information on the analysis of variance. Columns 7 and 8 contain the estimated variances for the unweighted and weighted estimates. Columns 9, 10, and 11 present the log of the ratio of the variances (unweighted/weighted), 95% confidence interval for the log of the ratio of the variances, and the ratio of the variances (unweighted/weighted). These figures show whether there is a statistically significant difference in the variance (precision) of the weighted and unweighted estimators and quantifies the size of the difference. If there was no difference between the unweighted variance and weighted variance, the log of the ratio of the variances would be zero. The ratio of the variances is a complementary way to quantify the differences. For example, **Table 3** shows that the ratio of the variances for the proportion of ANC clients who had no problem with the amount of time waited was 0.496. This figure means the weighted estimate – which has the larger variance – is using the information as if there is only 49.6% of the data or sample size available as compared to the unweighted estimate.

Columns 12-15 report on the analysis of the MSE, the measure which combines bias and variance. Columns 12 and 13 contain the MSE for the unweighted and weighted estimates. Columns 14 and 15 present the log of the ratio of the MSEs (unweighted/weighted) and the 95% confidence interval for the log of the ratio of the MSEs. These figures show whether there is a statistically significant difference in the MSE of the unweighted and weighted estimators. If there was no difference between the unweighted MSE and weighted MSE, the log of the ratio of the MSEs would be zero.

#### Bias

Of the ten TSPA indicators at the national level, the unweighted estimates showed a statistically significant bias for eight indicators. The remaining two indicators (Client had no problem with privacy from having others hear and Client had no problem with the cleanliness of the facility) showed no statistically significant bias. The bias ranged from an underestimation of the population truth by 3.6% to an overestimation of the population truth by 22.6%. Most indicators fell in the range of three to five percent bias.

Across the four TDHS indicators at the national level, the unweighted estimates showed a small but statistically significant bias for two indicators (ANC4 and Iron supplementation). The bias ranged from an underestimation of the population truth by 1.6% to 0.5%. For one indicator (ANC1) the weighted and unweighted estimates were the same, indicating no bias.

#### Variance

Of the ten TSPA indicators at the national level, the unweighted estimates showed a statistically significantly lower variance (relative to the weighted estimates) for eight indicators. The remaining two indicators (Provider asked about or performed syphilis test and ITNs or ITN vouchers available at the facility) showed no statistically significant differences in variance between weighted and unweighted estimates. However, there was a wide range of differences in variance between unweighted and weighted estimates. The ratio of the variances ranged from 0.310 to 0.984. This means for some indicators the weighted estimate used the information in the data as if there was only 31% of the data available as compared to the unweighted estimate while for other indicators the weighted estimate used almost all the data (98.4%).

For the TDHS indicators at the national level, the unweighted estimates showed a statistically significant lower variance for all four indicators. The ratio of the variances ranged from 0.681 to 0.730. This means for some indicators the weighted estimate used the information in the data as if there was 68% to 73% of the data available as compared to the unweighted estimate.

#### Mean squared error (MSE)

The trend across all indicators nationally was for the weighted estimates to be less biased and for the unweighted estimates to be less variable. Thus, it was useful to look at the combined effect of bias and variance as measured by the MSE to determine the preferred estimator. Across all TSPA indicators nationally, the weighted estimates showed a statistically significant lower MSE for eight out of the ten indicators while the unweighted estimates showed a statistically significantly lower MSE for two out of the ten indicators.

For the TDHS indicators at the national level, the weighted estimates showed a statistically significant lower MSE for two of the four indicators (ANC4+, Iron supplementation) while the unweighted estimates showed a statistically significantly lower MSE for the two of the four indicators (ANC1, IPTp).

For the indicators in which the MSE was lower for the weighted estimates, the effect of the bias trade-off was greater than the effect of the variance trade-off. For the indicators in which the MSE was lower for the unweighted estimates, there was no statistically significant bias for the unweighted estimates of these indicators (see Column 5). As such, the variance was driving the MSE in these cases, and the effect of the variance trade-off was greater than the effect of the bias trade-off.

### Q2: What is the best method for calculating standard errors?

#### Coverage probability

The coverage probability of a confidence interval is the proportion of the time that the interval contains the true value of interest. We would expect that for a nominal 95% confidence interval, the actual coverage probability would be close to 95%. A low coverage probability would be indicative of an anti-conservative standard error. A high coverage probability would be indicative of an overly conservative standard error.

**Figure 2:**
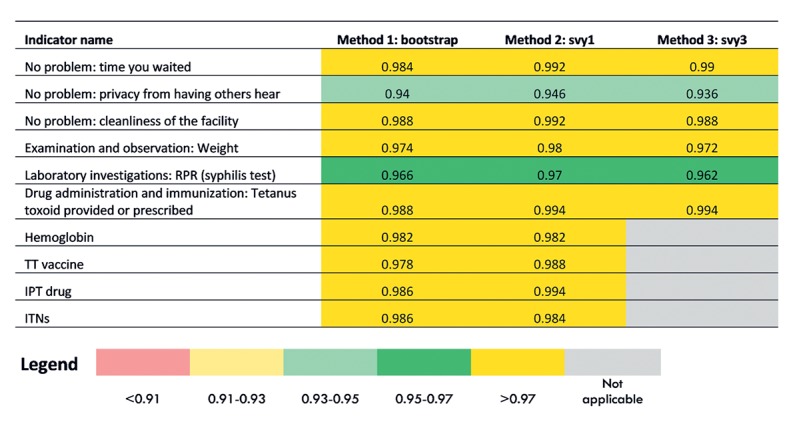
Coverage probability for each method for ten indicators, nationally.

**Figure 3:**
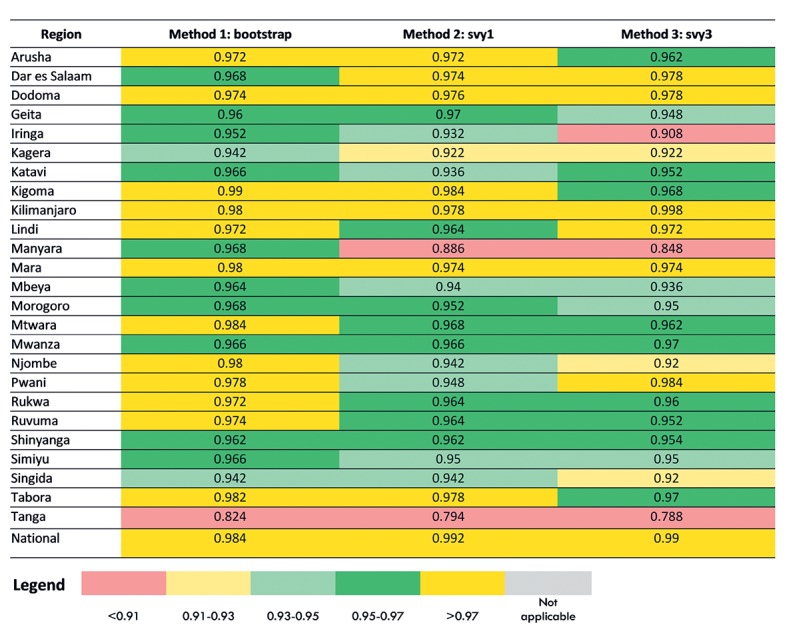
Coverage probability for each method for the facility survey indicator “client had no problem with the amount of time waited,” by region.

**Figure 2** shows the coverage probability for each of the three standard error estimation methods for the 10 TSPA indicators at the national level. **Figure 3** shows the same information for one indicator (Client had no problem with the amount of time waited) with regional disaggregation. Tables S14-S22 in **Online Supplementary Document** contain regional disaggregation for the other nine indicators. In each table, good coverage is indicated by cells colored in green. Light green indicates coverage probability between 0.93 and 0.95 and dark green shows coverage probability between 0.95 and 0.97. Fair coverage was indicated in each table by cells colored in yellow. Light yellow denotes coverage probability between 0.91 and 0.93 and dark yellow denotes coverage probability greater than 0.97. Poor coverage (less than 0.91) was indicated in each table by cells colored in red.

We found that the coverage probability results were similar across all three methods for generating standard errors (bootstrap, svy1, svy3) for the 10 TSPA indicators at the national level (**Figure 2**). For two indicators, the coverage probability was in the good range (one indicator dark green and one indicator light green). For the remaining eight indicators, the coverage probability was in the fair range (dark yellow). This indicated that for all three methods for most indicators, the standard errors were overly conservative. The three methods of generating standard errors appeared to be equal performers. However, when we examined the coverage probability for each indicator with regional disaggregation (**Figure 3**, Tables S14-S22 in **Online Supplementary Document**), we found greater variability in their performance. Although there was variability in performance of the three methods in the regional disaggregation, across all indicators the bootstrap method had the fewest instances of poor performance.

#### Average length of the confidence interval

A confidence interval gives us information about the uncertainty of an estimate. A narrower confidence interval indicates there is less uncertainty about the results. A wider confidence interval indicates there is more uncertainty about the results. As such, given the same coverage probability, a narrower confidence interval is desirable because it gives us more information about the true value for the population.

**Figure 4:**
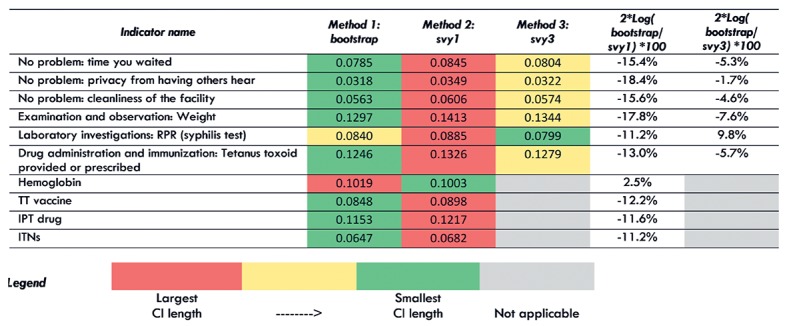
Average confidence interval (CI) length for each method for ten indicators, nationally.

**Figure 5:**
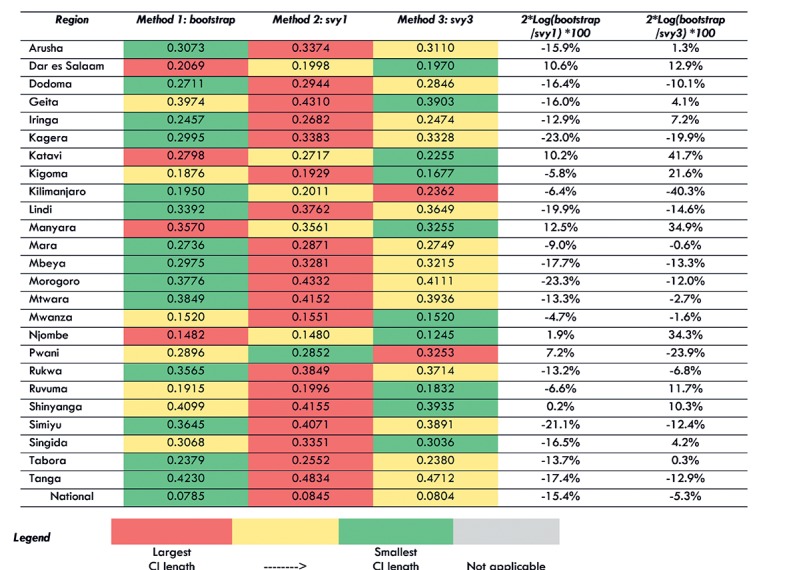
Average confidence interval (CI) length for each method for the facility survey indicator “client had no problem with the amount of time waited,” by region.

**Figure 4** shows the average confidence interval (CI) length for each of the three standard error estimation methods at the national level for the 10 TSPA indicators. **Figure 5** shows the same information for one indicator (Client had no problem with the amount of time waited) with regional disaggregation. Tables S23-S32 in **Online Supplementary Document** contain the regional disaggregation for the other nine indicators. The method with the smallest average CI length is colored green. The method with the largest average CI is colored red. The method with the average CI length falling in between the smallest and largest is colored yellow. The last two columns quantify the comparison between the bootstrap and the svy1 and svy3 methods. These figures can be interpreted as the equivalent percentage increase or decrease in information used by the bootstrap method as compared to the svy1 and svy3 methods.

We found that for eight out of 10 TSPA indicators nationally, the bootstrap method resulted in the smallest average CI length (**Figure 4**). The svy1 and svy3 methods each resulted in the smallest average CI length for one indicator. In addition, we found that the svy1 method resulted in the largest average CI length for 9 out of 10 indicators. Comparing the bootstrap method to the svy1 method, the bootstrap used 11.2% to 18.4% more information compared to svy1 method for nine indicators. However, for one indicator, the bootstrap method used 2.5% less information as compared to the svy1 method. Comparing the bootstrap method to the svy3 method, bootstrapping used 1.7% to 7.6% more information compared to the svy3 method for nine indicators. However, for one indicator, the bootstrap method used 9.8% less information compared to the svy3 method.

When we examined the average CI length for each indicator with regional disaggregation (**Figure 5**, Tables S23-31 in **Online Supplementary Document**) we found greater variability in the performance of the three methods for generating standard errors. Although there was increased variability in performance in the regional disaggregation, the bootstrap method had the fewest instances of largest CI length across all indicators and the most instances of smallest CI length. We also found that the svy1 method had the most instances of largest CI length and the fewest instances of smallest CI length.

## DISCUSSION

This applied study investigated the effect of survey weights on the bias and variance of point estimates using data from the 2015 TDHS and TSPA surveys. We also evaluated multiple methods for variance estimation to determine the best estimator of the standard error for the TSPA. Our results showed that while weighted estimates were less biased, unweighted estimates were less variable. This finding is consistent with what we would expect based on statistical theory [12]. Further, we found that for the majority of TDHS and TSPA indicators assessed, the weighted estimates had a lower MSE. The lower MSE indicated that the benefit of avoiding bias by using weights was greater than the cost of increased variance. The simple answer to our first research question – to weight or not to weight when estimating rates in these two surveys – is to use the survey weights. Our results also showed that the best performer for variance estimation for TSPA data was the cluster bootstrap method. The second-best performer was the design-based svy method with 3 levels. The design-based svy method with one level was the worst performer for most indicators. The simple answer to our second research question– the best method for calculating standard errors – is to use the cluster bootstrap method.

The trade-off between bias and variance for complex survey data are well known [5]. Statisticians recommend assessing the efficiency of performing a weighted analysis, for example using the MSE, to determine if the trade-off is worthwhile [13,14]. In this analysis, we took this approach and found that weighted estimates, which gave an unbiased population estimate, were generally more efficient than unweighted estimates. For some indicators, the unweighted estimate resulted in a large bias, such as for the indicator “hemoglobin test available at the facility” from the TSPA data. For this indicator, the national unweighted simulation estimate was 52.0% while the weighted simulation estimate was 29.4% and the true population rate was 29.6%. One explanation for this finding may be that laboratory capacity is often only available at higher level facilities, which are oversampled in the SPA. Unweighted estimates do not account for this oversampling, resulting in significant bias in this unweighted simulation estimate. However, we did find that the weighted and unweighted simulation estimates were not very different for several indicators, and for these indicators the unweighted estimate had a small, negligible bias. For example, for the TSPA indicator “client had no problem with the cleanliness of the facility,” the national unweighted simulation estimate was 85.7% while the weighted simulation estimate was 85.4%, and the weighted true estimate was 85.3%. In addition, we found that for some indicators there was no bias at all. For example, for the TDHS indicator ANC1, the national unweighted simulation estimate, weighted simulation estimate, and weighted true estimate were all 98.0%. The lack of variability in the ANC1 indicator may account for the absence of bias in this indicator. Although these results show greater efficiency overall for weighted estimates, they also suggest that for some indicators an unweighted analysis may produce less error overall. Typically, when analyzing complex survey data, the same analytical methods will be applied to all indicators. As such, when determining the methods to employ, we must look at the totality of the results obtained from a study such as this one and decide on the best methods to apply for all indicators.

Methods of variance estimation for complex surveys include Taylor series linearization as well as resampling techniques such as the jackknife, the bootstrap, and balanced repeated replication [15-17]. In this analysis, we applied both linearization (svy1 and svy3) and a resampling technique (bootstrap) to generate evidence on which method performs the best. Although we determined that the bootstrap method was the best performer (most indicators with coverage probability between 0.93 and 0.97), we did not find large differences in performance between the different methods of variance estimation. As such, it may not be necessary to invest time in determining the best method for variance estimation in every circumstance. For the NEP evaluation in Tanzania, several factors in addition to the performance of the different variance estimation methods helped to determine which method of variance estimation to implement. Another study in Tanzania found that policy-makers have a limited understanding of confidence intervals and the concept of variance, making this information of limited use for decision-making [18]. The target audience for the NEP evaluation was government officials who are generally more interested in point estimates than the uncertainty around those estimates. Therefore, the small differences in variance obtained from the three variance estimation methods explored in this analysis were not particularly relevant. In addition, the NEP evaluation assessed changes over time in the quality of care at health facilities utilizing the SPA surveys from 2006 and 2015. However, the 2006 TSPA data set did not include the information required to generate the fpc at all levels, which is required for the design-based svy3 method. As a result, the bootstrap and design-based svy1 methods were the only feasible options with these data sets. Finally, the NEP Tanzania team had limited statistical capacity. The team felt more comfortable with the design-based svy methods that could be easily implemented in statistical software they were familiar with. Based on all these considerations, the NEP evaluation chose to use the svy1 method for generating standard errors.

This study has some limitations. Our analysis focused on a set of indicators from two surveys in Tanzania which may not be more broadly applicable to all indicators nor across countries. However, these findings are useful for anyone using TDHS and TSPA data and can serve as a useful example for groups seeking to analyze complex survey data in other settings. In addition, we were not able to investigate the methods for standard error estimation using TDHS data due to computational limitations. Finally, our analysis also did not include all possible variance estimation methods. Several replication methods were excluded such as jackknife and balanced repeated replication. However, the most common methods used at country level were included in the analysis. To assist others who would like to implement the methods and/or replicate this study with a different data set, the statistical code written for these analyses is publicly available: http://doi.org/10.5281/zenodo.1311839.

## CONCLUSION

As complex survey data become more widely used in LMICs, there is a need for guidance on the methods for analysis of these data. Although the standard practice has been to conduct a design-based analysis using survey weights and the single-level svy method for calculating standard errors, this study puts forth an alternative approach for assessing whether analyses should be weighted as well as for selecting a variance estimation method. As these analyses were specific to only two surveys from Tanzania, there is a need to replicate them in other settings, as well as with other complex survey data sets to obtain more generalizable guidance on analytical methods for complex survey data in LMICs.

## Additional material

Online Supplementary Document
