# Supplementary material for: Methods for analysis of complex survey data: an application using the Tanzanian 2015 Demographic and Health Survey and Service Provision Assessment
Source: J Glob Health. 2019 Dec 16;9(2):020902. doi: 10.7189/jogh.09.020902 (PMC6925968; doi:10.7189/jogh.09.020902)
Supplement: Online Supplementary Document [file jogh-09-020902-s001.pdf]

Supplementary tables

Supplementary Table 1: Characteristics of indicators selected for the analysis

Indicator	National	Regional Min.	Regional Max.	Number of observations
WOMEN				
ANC1[1]	98.0%	93.3%	100.0%	6,078
ANC4+[2]	50.9%	21.1%	75.0%	6,078
IPTp[3]	35.8%	17.7%	50.0%	6,078
Iron supplementation[4]	21.8%	7.2%	32.8%	6,078
ALL ANC CLIENTS				
Client had no problem with the amount of time waited	70.0%	45.2%	91.6%	3,641
Client had no problem with privacy from having others hear	85.3%	65.7%	98.7%	3,641
Client had no problem with the cleanliness of the facility	96.8%	85.3%	99.8%	3,641
ANC CLIENTS ATTENDING FOR FIRST VISIT				
Provider weighed the client	13.7%	0.0%	67.8%	1,607
Provider asked about or performed syphilis test	53.5%	20.8%	82.7%	1,607
Provider provided or prescribed tetanus toxoid vaccine	80.8%	45.5%	100.0%	1,607
HEALTH FACILITIES				
Hemoglobin test available at the facility	9.8%	1.4%	30.3%	949
TT vaccine available at the facility	29.5%	9.4%	89.0%	949
IPT drug available at the facility	63.2%	27.0%	99.5%	949
ITNs or ITN vouchers available at the facility	86.4%	65.4%	100.0%	949

Note: ANC = antenatal care, IPTp = Intermittent Preventive Treatment of malaria during pregnancy, TT = tetanus toxoid, ITN= insecticide treated bed-net

[1]ANC1 definition - Percentage of women ages 15-49 with a live birth in the 5 years preceding the survey who were attended at least once during pregnancy
[2]Percentage of women ages 15-49 with a live birth in the 5 years preceding the survey who were attended at least four times during pregnancy
[3]Percentage of women ages 15-49 with a live birth in the 5 years preceding the survey who received two or more doses of a sulfadoxine-pyrimethamine to prevent malaria during their last pregnancy
[4]Percentage of women ages 15-49 with a live birth in the 5 years preceding the survey who took iron supplementation for 90+ days

Supplementary Table 2: Comparison of bias, variance, and MSE for weighted vs. unweighted estimates for the household survey indicator "ANC1 coverage", by region

				**BIAS**		**VARIANCE**					**MEAN SQUARED ERROR (MSE)**			
Column 1	2	3	4	5	6	7	8	9	10	11	12	13	14	15
Region	Weighted TRUTH (x 10^2)	Average Unweighted Simulation Estimate (x 10^2)	Average Weighted Simulation Estimate (x 10^2)	Mean Difference in Bias (x 10^2)	95% CI Mean Difference in Bias	Variance of Unweighted Estimate (x 10^3)	Variance of Weighted Estimate (x 10^3	Log of the Ratio of the Variances (Unweighted/ Weighted)	95% CI of Log of the Ratio of the Variances	Ratio of the Variances (Unweighted/ Weighted)	MSE of Unweighted Estimates (x 10^3)	MSE of Weighted Estimates (x 10^3)	Log of the Ratio of the MSEs (Unweighted/ Weighted)	95% CI of Log of the Ratio of the MSEs
Arusha	94.5	94.5	94.5	0	(-1, 0.9)	0.3	0.3	0.041	(-0.005, 0.088)	1.042	0.3	0.3	0.063	(-0.011, 0.073)
Dar es Salaam	97.6	97.6	97.6	0	(-0.5, 0.5)	0.1	0.1	0	(-0.009, 0.093)	1.000	0.1	0.1	0.044*	(0.041, 0.111)
Dodoma	99.2	99.0	99.2	-0.2*	(-0.7, 0)	0.1	0.0	0.403*	(0.371, 0.463)	1.496	0.1	0.0	0.468*	(0.427, 0.508)
Geita	96.8	96.7	96.7	-0.1	(-0.7, 0.5)	0.3	0.3	-0.116*	(-0.154, -0.099)	0.890	0.3	0.3	-0.135*	(-0.136, -0.054)
Iringa	99.4	99.4	99.4	0	(-0.1, 0.2)	0.0	0.0	-0.069*	(-0.093, -0.05)	0.933	0.0	0.0	-0.068*	(-0.073, -0.054)
Kagera	99.5	99.6	99.5	0	(0, 0.2)	0.0	0.0	-0.174*	(-0.198, -0.154)	0.840	0.0	0.0	-0.161*	(-0.174, -0.122)
Katavi	97.2	96.2	97.1	-0.9*	(-2.1, -0.1)	0.2	0.1	0.506*	(0.47, 0.571)	1.658	0.3	0.1	0.691*	(0.579, 0.702)
Kigoma	99.6	99.7	99.6	0.1	(0, 0.3)	0.0	0.0	-0.373*	(-0.381, -0.273)	0.688	0.0	0.0	-0.284*	(-0.282, -0.197)
Kilimanjaro	98.3	98.5	98.4	0.1	(-0.3, 0.7)	0.1	0.1	-0.175*	(-0.215, -0.136)	0.839	0.1	0.1	-0.168*	(-0.169, -0.121)
Lindi	99.1	98.9	99.1	-0.2	(-0.5, 0.1)	0.1	0.0	0.244*	(0.241, 0.318)	1.276	0.1	0.0	0.306*	(0.298, 0.376)
Manyara	98.2	99.2	98.3	0.9*	(0, 1.8)	0.0	0.1	-1.283*	(-1.315, -1.189)	0.277	0.1	0.1	-0.264*	(-0.312, -0.202)
Mara	97.1	96.8	97.1	-0.2	(-0.6, 0.1)	0.2	0.2	0.056*	(0.005, 0.062)	1.058	0.2	0.2	0.068*	(0.022, 0.067)
Mbeya	97.7	98.0	97.9	0.1	(-0.3, 0.7)	0.2	0.2	-0.142*	(-0.187, -0.125)	0.867	0.2	0.2	-0.133*	(-0.248, -0.13)
Morogoro	99.2	99.5	99.2	0.3	(0, 0.9)	0.0	0.1	-0.786*	(-0.799, -0.723)	0.456	0.0	0.1	-0.642*	(-0.662, -0.51)
Mtwara	100.0	100.0	100.0	0	(0, 0)	0.0	0.0	-1.054*	(-2.109, -0.978)	0.348	0.0	0.0	-1.4*	(-2.503, -1.192)
Mwanza	93.3	92.9	93.2	-0.3	(-1.5, 1)	0.3	0.4	-0.257*	(-0.262, -0.16)	0.773	0.4	0.4	-0.198*	(-0.21, -0.128)
Njombe	99.0	98.9	99.0	0	(-0.2, 0.1)	0.1	0.1	0.047*	(0.017, 0.051)	1.048	0.1	0.1	0.036*	(0.035, 0.051)
Pwani	99.4	99.5	99.4	0.1*	(0, 0.3)	0.0	0.0	-0.285*	(-0.286, -0.245)	0.752	0.0	0.0	-0.26*	(-0.282, -0.236)
Rukwa	96.3	96.0	96.3	-0.4	(-0.9, 0.2)	0.1	0.1	0.045*	(0.049, 0.129)	1.046	0.1	0.1	0.141*	(0.026, 0.15)
Ruvuma	99.7	99.6	99.7	-0.1	(-0.4, 0)	0.0	0.0	0.522*	(0.48, 0.575)	1.685	0.0	0.0	0.557*	(0.552, 0.602)
Shinyanga	98.1	98.1	98.2	-0.1	(-0.2, 0.2)	0.1	0.1	0.026*	(0.006, 0.052)	1.026	0.1	0.1	0.03*	(0.005, 0.034)
Simiyu	98.5	98.7	98.5	0.2	(-0.2, 0.7)	0.0	0.0	-0.324*	(-0.388, -0.269)	0.723	0.0	0.0	-0.245*	(-0.263, -0.087)
Singida	99.2	99.2	99.1	0	(-0.2, 0.3)	0.0	0.0	-0.065*	(-0.127, -0.044)	0.937	0.0	0.0	-0.093*	(-0.096, -0.016)
Tabora	98.0	97.9	98.0	0	(-0.2, 0.2)	0.0	0.0	0.027	(-0.012, 0.04)	1.027	0.0	0.0	0.01	(-0.021, 0.033)
Tanga	99.4	99.0	99.3	-0.3	(-0.8, 0)	0.0	0.0	0.5*	(0.429, 0.579)	1.649	0.1	0.0	0.63*	(0.554, 0.697)
National	98.0	98.0	98.0	0	(-0.2, 0.3)	0.0	0.0	-0.384*	(-0.415, -0.222)	0.681	0.0	0.0	-0.292*	(-0.294, -0.124)

Note: * indicates p-value <0.05, MSE = mean squared error, CI = confidence interval

Supplementary Table 3: Comparison of bias, variance, and MSE for weighted vs. unweighted estimates for the household survey indicator "IPTp coverage", by region

				BIAS		VARIANCE					MEAN SQUARED ERROR (MSE)			
Column 1	2	3	4	5	6	7	8	9	10	11	12	13	14	15
Region	Weighted TRUTH (x 10^2)	Average Unweighted Simulation Estimate (x 10^2)	Average Weighted Simulation Estimate (x 10^2)	Mean Difference in Bias (x 10^2)	95% CI Mean Difference in Bias	Variance of Unweighted Estimate (x 10^3)	Variance of Weighted Estimate (x 10^3	Log of the Ratio of the Variances (Unweighted/ Weighted)	95% CI of Log of the Ratio of the Variances	Ratio of the Variances (Unweighted/ Weighted)	MSE of Unweighted Estimates (x 10^3)	MSE of Weighted Estimates (x 10^3)	Log of the Ratio of the MSEs (Unweighted/ Weighted)	95% CI of Log of the Ratio of the MSEs
Arusha	34.2	33.2	34.2	-1	(-2.7, 0.8)	2.0	2.2	-0.114*	(-0.13, -0.071)	0.892	2.1	2.2	-0.07*	(-0.074, -0.011)
Dar es Salaam	45.3	45.4	45.2	0.2	(-1.5, 1.8)	0.7	0.8	-0.063*	(-0.115, -0.009)	0.939	0.7	0.8	-0.063*	(-0.114, -0.029)
Dodoma	33.8	34.5	33.7	0.8	(-1.2, 3.3)	2.2	2.3	-0.029*	(-0.096, -0.014)	0.971	2.3	2.3	-0.05*	(-0.069, -0.019)
Geita	17.7	18.9	17.8	1.1	(-0.1, 2.8)	0.7	0.6	0.258*	(0.2, 0.296)	1.294	0.9	0.6	0.305*	(0.266, 0.367)
Iringa	42.4	43.9	42.4	1.6	(-0.1, 4)	1.5	1.3	0.087*	(0.073, 0.184)	1.091	1.7	1.3	0.198*	(0.13, 0.212)
Kagera	48.7	49.6	48.9	0.7	(-1.1, 3)	1.8	2.0	-0.115*	(-0.172, -0.098)	0.891	1.9	2.0	-0.104*	(-0.24, -0.1)
Katavi	23.9	23.1	23.7	-0.6	(-2.5, 1.3)	0.8	0.9	-0.084	(-0.102, 0.006)	0.920	0.9	0.9	-0.029	(-0.066, 0.045)
Kigoma	27.1	28.4	27.7	0.7	(-4.1, 5.3)	2.0	2.9	-0.256*	(-0.434, -0.282)	0.774	2.2	2.9	-0.379*	(-0.38, -0.279)
Kilimanjaro	37.6	38.5	37.7	0.8	(-1, 3.1)	1.6	1.5	0.023*	(0.006, 0.109)	1.023	1.6	1.5	0.075*	(0.014, 0.09)
Lindi	36.3	38.0	36.6	1.4	(-0.1, 3)	1.1	0.9	0.253*	(0.149, 0.233)	1.288	1.3	0.9	0.331*	(0.327, 0.453)
Manyara	37.4	38.6	37.8	0.9	(-2.3, 3.9)	1.4	1.6	-0.137*	(-0.185, -0.053)	0.872	1.5	1.6	-0.083*	(-0.236, -0.081)
Mara	31.9	33.3	32.3	1	(-1, 2.9)	1.4	1.5	-0.082*	(-0.125, -0.028)	0.922	1.6	1.5	-0.001	(-0.013, 0.143)
Mbeya	31.8	32.6	32.0	0.5	(-1.7, 2.1)	1.0	1.4	-0.326*	(-0.399, -0.297)	0.722	1.0	1.4	-0.312*	(-0.318, -0.165)
Morogoro	49.8	48.6	50.0	-1.3	(-2.9, 0)	1.4	1.3	0.119*	(0.087, 0.157)	1.126	1.6	1.3	0.206*	(0.199, 0.3)
Mtwara	42.5	42.7	42.6	0	(-0.8, 1)	1.7	1.5	0.107*	(0.097, 0.13)	1.113	1.7	1.5	0.113*	(0.09, 0.113)
Mwanza	27.2	30.4	27.4	3	(-0.3, 6.8)	2.3	2.4	-0.088	(-0.095, 0.034)	0.916	3.3	2.4	0.192*	(0.035, 0.209)
Njombe	36.6	37.0	36.8	0.2	(-0.8, 1.3)	1.2	1.2	-0.067*	(-0.085, -0.032)	0.935	1.2	1.2	-0.05*	(-0.052, -0.033)
Pwani	43.2	43.3	43.5	-0.2	(-1.7, 1.2)	1.9	1.8	0.073*	(0.045, 0.102)	1.076	1.9	1.8	0.061*	(0.051, 0.086)
Rukwa	39.2	38.5	39.1	-0.6	(-2.9, 1.5)	1.6	2.0	-0.248*	(-0.286, -0.199)	0.781	1.7	2.0	-0.197*	(-0.252, -0.18)
Ruvuma	30.1	31.4	30.5	0.9	(-1.2, 3.2)	1.6	1.7	-0.107*	(-0.133, -0.035)	0.898	1.8	1.8	-0.035*	(-0.131, -0.02)
Shinyanga	43.7	41.1	43.6	-2.5*	(-5.8, -0.3)	1.7	2.4	-0.341*	(-0.383, -0.294)	0.711	2.4	2.4	-0.187*	(-0.195, -0.058)
Simiyu	28.3	29.1	28.6	0.5	(-1.9, 2.3)	0.5	0.8	-0.324*	(-0.453, -0.291)	0.723	0.6	0.8	-0.325*	(-0.333, -0.177)
Singida	39.2	41.0	39.3	1.7	(-2.1, 5.5)	1.9	2.7	-0.301*	(-0.38, -0.256)	0.740	2.3	2.7	-0.254*	(-0.302, -0.231)
Tabora	19.7	20.1	19.8	0.2	(-0.5, 1)	0.9	1.0	-0.076*	(-0.092, -0.047)	0.927	1.0	1.0	-0.063	(-0.068, 0.028)
Tanga	50.0	47.0	49.7	-2.7	(-6.1, 0.2)	2.0	2.2	-0.117*	(-0.154, -0.038)	0.889	2.8	2.2	0.126*	(0.067, 0.171)
National	35.8	35.3	35.8	-0.5	(-1.4, 0.3)	0.1	0.1	-0.345*	(-0.435, -0.27)	0.708	0.1	0.1	-0.159*	(-0.276, -0.117)

Note: * indicates p-value <0.05, MSE = mean squared error, CI = confidence interval

Supplementary Table 4: Comparison of bias, variance, and MSE for weighted vs. unweighted estimates for the household survey indicator “Iron supplementation coverage”, by region

				BIAS		VARIANCE					MEAN SQUARED ERROR (MSE)			
Column 1	2	3	4	5	6	7	8	9	10	11	12	13	14	15
Region	Weighted TRUTH (x 10^2)	Average Unweighted Simulation Estimate (x 10^2)	Average Weighted Simulation Estimate (x 10^2)	Mean Difference in Bias (x 10^2)	95% CI Mean Difference in Bias	Variance of Unweighted Estimate (x 10^3)	Variance of Weighted Estimate (x 10^3	Log of the Ratio of the Variances (Unweighted/ Weighted)	95% CI of Log of the Ratio of the Variances	Ratio of the Variances (Unweighted/ Weighted)	MSE of Unweighted Estimates (x 10^3)	MSE of Weighted Estimates (x 10^3)	Log of the Ratio of the MSEs (Unweighted/ Weighted)	95% CI of Log of the Ratio of the MSEs
Arusha	18.7	18.1	18.8	-0.7	(-2, 0.5)	0.8	0.8	0.104*	(0.038, 0.12)	1.109	0.9	0.8	0.103*	(0.071, 0.142)
Dar es Salaam	29.3	28.0	29.2	-1.2	(-3.6, 0.8)	0.9	1.2	-0.345*	(-0.386, -0.285)	0.708	1.0	1.2	-0.244*	(-0.304, -0.161)
Dodoma	27.3	27.7	27.5	0.2	(-1, 1.3)	1.1	1.2	-0.058*	(-0.109, -0.047)	0.943	1.2	1.2	-0.067*	(-0.083, -0.043)
Geita	17.3	17.4	17.3	0.1	(-1.3, 1.4)	0.7	0.7	0.03	(-0.006, 0.084)	1.030	0.7	0.7	0.03	(-0.026, 0.032)
Iringa	30.4	31.8	30.2	1.6	(-0.8, 4.5)	2.5	2.0	0.194*	(0.141, 0.234)	1.214	2.7	2.0	0.222*	(0.106, 0.225)
Kagera	12.2	13.0	12.2	0.9	(0, 2)	0.9	0.8	0.154*	(0.124, 0.184)	1.167	1.0	0.8	0.197*	(0.059, 0.219)
Katavi	12.6	12.4	12.6	-0.2	(-1.4, 0.9)	0.4	0.4	-0.01	(-0.061, 0.027)	0.990	0.4	0.4	-0.003	(-0.11, 0.001)
Kigoma	7.2	8.1	7.3	0.8	(-0.7, 2.5)	0.5	0.3	0.476*	(0.366, 0.523)	1.610	0.6	0.3	0.468*	(0.415, 0.479)
Kilimanjaro	28.7	29.6	29.0	0.6	(-1.1, 2.5)	2.1	2.1	-0.004	(-0.017, 0.059)	0.996	2.2	2.1	0.034	(-0.029, 0.04)
Lindi	32.8	32.1	32.7	-0.6	(-3.5, 2.9)	2.9	3.7	-0.285*	(-0.318, -0.23)	0.752	2.9	3.7	-0.258*	(-0.29, -0.25)
Manyara	11.0	11.4	11.1	0.3	(-2.2, 2.6)	0.6	0.9	-0.355*	(-0.459, -0.322)	0.701	0.6	0.9	-0.363*	(-0.44, -0.361)
Mara	9.2	8.7	9.1	-0.4	(-1, 0.2)	0.3	0.3	0.038	(-0.003, 0.067)	1.038	0.3	0.3	0.072	(-0.066, 0.098)
Mbeya	32.0	33.0	32.0	1	(-1.5, 3.9)	1.1	1.3	-0.189*	(-0.309, -0.175)	0.827	1.2	1.3	-0.169*	(-0.173, -0.097)
Morogoro	28.6	27.7	28.6	-0.9	(-3.7, 1.2)	0.7	0.9	-0.163*	(-0.299, -0.159)	0.850	0.8	0.9	-0.198*	(-0.221, -0.141)
Mtwara	27.4	27.3	27.3	-0.1	(-0.7, 0.8)	1.5	1.4	0.119*	(0.088, 0.126)	1.126	1.5	1.4	0.113*	(0.098, 0.122)
Mwanza	19.4	20.3	19.5	0.8	(-1.1, 3.2)	0.6	0.6	-0.08	(-0.108, 0.043)	0.923	0.7	0.6	0.013*	(0.008, 0.109)
Njombe	27.1	27.3	27.4	-0.1	(-1.5, 1.5)	1.7	1.8	-0.038*	(-0.073, -0.013)	0.963	1.7	1.8	-0.058*	(-0.081, -0.055)
Pwani	25.2	25.1	25.0	0.2	(-0.9, 1)	0.9	0.8	0.124*	(0.065, 0.127)	1.132	0.9	0.8	0.098*	(0.064, 0.097)
Rukwa	22.4	21.9	22.5	-0.6	(-1.9, 0.7)	0.6	0.6	-0.011	(-0.025, 0.069)	0.989	0.6	0.6	0.069	(-0.117, 0.074)
Ruvuma	21.6	23.1	21.9	1.2*	(0.2, 2.5)	1.1	1.0	0.074*	(0.071, 0.138)	1.077	1.4	1.0	0.175*	(0.072, 0.202)
Shinyanga	25.8	25.8	25.8	-0.1	(-1.2, 0.9)	1.0	1.0	0.001	(-0.012, 0.046)	1.001	1.0	1.0	0.023*	(0.009, 0.035)
Simiyu	8.8	10.1	8.9	1.1	(-0.1, 2.3)	0.4	0.4	0.039	(-0.07, 0.054)	1.040	0.5	0.4	0.199*	(0.189, 0.337)
Singida	21.9	23.3	21.8	1.5	(-1.7, 4.6)	0.7	1.4	-0.674*	(-0.749, -0.621)	0.509	0.9	1.4	-0.569*	(-0.664, -0.48)
Tabora	21.9	21.4	22.0	-0.6	(-1.4, 0.1)	1.2	1.2	0.016	(0, 0.043)	1.016	1.2	1.2	0.009	(-0.085, 0.015)
Tanga	29.2	29.5	29.0	0.6	(-1.1, 2.3)	0.7	0.8	-0.159*	(-0.166, -0.063)	0.853	0.7	0.8	-0.112*	(-0.117, -0.027)
National	21.8	20.9	21.8	-0.9*	(-1.7, -0.2)	0.0	0.1	-0.315*	(-0.416, -0.233)	0.730	0.1	0.1	0.392*	(0.357, 0.56)

Note: * indicates p-value <0.05, MSE = mean squared error, CI = confidence interval

Supplementary Table 5: Comparison of bias, variance, and MSE for weighted vs. unweighted estimates for the indicator "Client had no problem with privacy from having others hear", by region

				BIAS		VARIANCE					MEAN SQUARED ERROR (MSE)			
Column 1	2	3	4	5	6	7	8	9	10	11	12	13	14	15
Region	Weighted TRUTH (x 10^2)	Average Unweighted Simulation Estimate (x 10^2)	Average Weighted Simulation Estimate (x 10^2)	Mean Difference in Bias (x 10^2)	95% CI Mean Difference in Bias	Variance of Unweighted Estimate (x 10^3)	Variance of Weighted Estimate (x 10^3	Log of the Ratio of the Variances (Unweighted/ Weighted)	95% CI of Log of the Ratio of the Variances	Ratio of the Variances (Unweighted/ Weighted)	MSE of Unweighted Estimates (x 10^3)	MSE of Weighted Estimates (x 10^3)	Log of the Ratio of the MSEs (Unweighted/ Weighted)	95% CI of Log of the Ratio of the MSEs
Arusha	98.6	98.0	98.6	-0.6	(-1.6, 0)	0.1	0.1	0.757*	(0.578, 0.732)	2.132	0.1	0.1	0.85*	(0.841, 1.005)
Dar es Salaam	96.5	97.3	96.6	0.7	(-2, 4.7)	0.1	0.5	-1.703*	(-1.787, -1.531)	0.182	0.2	0.5	-1.417*	(-1.43, -1.182)
Dodoma	99.6	99.2	99.6	-0.4	(-1.3, 0)	0.1	0.0	1.414*	(1.351, 1.436)	4.113	0.1	0.0	1.584*	(1.468, 1.583)
Geita	87.6	90.5	88.0	2.5	(-3.3, 10.1)	3.0	6.6	-0.831*	(-0.85, -0.725)	0.436	3.9	6.6	-0.638*	(-0.684, -0.574)
Iringa	98.0	96.6	98.0	-1.4*	(-3.6, 0)	0.5	0.2	0.857*	(0.772, 0.919)	2.356	0.7	0.2	1.035*	(0.989, 1.191)
Kagera	99.8	99.5	99.8	-0.3	(-1, 0)	0.0	0.0	1.496*	(1.368, 1.563)	4.465	0.0	0.0	1.64*	(1.546, 1.69)
Katavi	98.8	99.2	98.8	0.4	(0, 1.3)	0.1	0.1	-0.769*	(-0.801, -0.729)	0.463	0.1	0.1	-0.613*	(-0.69, -0.597)
Kigoma	99.8	99.6	99.8	-0.3	(-0.9, 0)	0.0	0.0	1.756*	(1.67, 1.828)	5.791	0.0	0.0	1.971*	(1.916, 2.115)
Kilimanjaro	85.3	88.7	85.2	3.5	(-0.1, 11.1)	1.4	3.1	-0.676*	(-0.894, -0.669)	0.508	2.6	3.1	-0.412*	(-0.616, -0.338)
Lindi	97.1	97.7	97.0	0.7	(-0.9, 3.3)	0.3	0.5	-0.762*	(-0.822, -0.67)	0.467	0.3	0.5	-0.743*	(-0.99, -0.733)
Manyara	99.7	99.3	99.7	-0.4	(-1.4, 0)	0.0	0.0	1.881*	(1.705, 1.884)	6.559	0.1	0.0	2.02*	(1.883, 2.141)
Mara	98.4	96.7	98.4	-1.7*	(-3.3, -0.5)	0.3	0.1	0.705*	(0.553, 0.75)	2.023	0.6	0.1	1.132*	(1.133, 1.3)
Mbeya	97.2	95.8	96.9	-1.2	(-4.6, 3.7)	0.2	0.5	-0.993*	(-1.194, -0.808)	0.371	0.4	0.5	-0.542*	(-0.703, -0.512)
Morogoro	96.5	97.2	96.6	0.6	(-1.6, 3.5)	0.2	0.5	-0.853*	(-0.941, -0.752)	0.426	0.3	0.5	-0.785*	(-0.829, -0.486)
Mtwara	92.9	92.3	93.2	-0.9	(-3.7, 1.1)	2.6	1.8	0.357*	(0.322, 0.418)	1.428	2.6	1.8	0.362*	(0.303, 0.373)
Mwanza	95.7	97.9	95.7	2.2	(-1, 6.1)	0.1	0.6	-1.796*	(-1.936, -1.718)	0.166	0.6	0.6	-0.527*	(-0.533, -0.277)
Njombe	95.3	98.1	95.8	2.4	(0, 7.2)	0.3	1.6	-1.573*	(-1.618, -1.489)	0.207	1.2	1.6	-0.718*	(-0.803, -0.674)
Pwani	98.4	97.0	98.3	-1.3*	(-3.5, 0)	0.4	0.1	1.016*	(0.918, 1.136)	2.761	0.6	0.1	1.221*	(1.198, 1.616)
Rukwa	93.5	91.2	93.5	-2.3	(-8.3, 2.8)	1.5	1.0	0.478*	(0.326, 0.558)	1.614	2.0	1.0	0.619*	(0.532, 0.834)
Ruvuma	98.7	97.6	98.7	-1.1*	(-2.8, 0)	0.2	0.1	1.184*	(1.037, 1.176)	3.266	0.4	0.1	1.342*	(1.275, 1.364)
Shinyanga	96.4	98.5	96.6	1.9*	(0, 6.7)	0.2	1.2	-1.591*	(-1.695, -1.546)	0.204	0.7	1.2	-0.933*	(-1.209, -0.936)
Simiyu	96.5	97.0	96.6	0.4	(-2, 3)	0.2	0.4	-0.82*	(-0.872, -0.643)	0.441	0.2	0.4	-0.72*	(-0.906, -0.716)
Singida	89.6	96.1	89.8	6.38	(0, 17.3)	1.1	6.3	-1.722*	(-1.804, -1.648)	0.179	5.2	6.3	-0.663*	(-0.742, -0.321)
Tabora	98.5	98.8	98.5	0.3	(-0.5, 1.4)	0.1	0.1	-0.639*	(-0.738, -0.552)	0.528	0.1	0.1	-0.581*	(-0.58, -0.441)
Tanga	96.9	97.8	96.9	0.9	(-1.6, 5)	0.2	0.7	-1.314*	(-1.306, -1.077)	0.269	0.3	0.7	-0.949*	(-1.134, -0.953)
National	96.8	96.8	96.8	0	(-0.6, 0.7)	0.0	0.0	-0.536*	(-0.726, -0.518)	0.585	0.0	0.0	-0.595*	(-0.69, -0.553)

Note: * indicates p-value <0.05, MSE = mean squared error, CI = confidence interval

Supplementary Table 6: Comparison of bias, variance, and MSE for weighted vs. unweighted estimates for the indicator "Client had no problem with the cleanliness of the facility", by region

	BIAS	VARIANCE	MEAN SQUARED ERROR (MSE)

Column 1	2	3	4	5	6	7	8	9	10	11	12	13	14	15
Region	Weighted TRUTH (x 10^2)	Average Unweighted Simulation Estimate (x 10^2)	Average Weighted Simulation Estimate (x 10^2)	Mean Difference in Bias (x 10^2)	95% CI Mean Difference in Bias	Variance of Unweighted Estimate (x 10^3)	Variance of Weighted Estimate (x 10^3	Log of the Ratio of the Variances (Unweighted/ Weighted)	95% CI of Log of the Ratio of the Variances	Ratio of the Variances (Unweighted/ Weighted)	MSE of Unweighted Estimates (x 10^3)	MSE of Weighted Estimates (x 10^3)	Log of the Ratio of the MSEs (Unweighted/ Weighted)	95% CI of Log of the Ratio of the MSEs
Arusha	83.3	85.3	83.7	1.6	(-3.2, 6.7)	1.5	1.6	-0.091	(-0.174, 0.025)	0.913	1.9	1.6	0.077*	(0.058, 0.197)
Dar es Salaam	90.6	94.4	90.5	3.9	(-1.7, 10.8)	0.3	1.5	-1.691*	(-1.805, -1.498)	0.184	1.8	1.5	-0.365*	(-0.401, -0.237)
Dodoma	91.3	89.8	91.5	-1.7	(-6.4, 2.7)	1.4	1.4	0.016	(-0.119, 0.068)	1.016	1.6	1.4	0.054	(-0.018, 0.116)
Geita	80.5	75.4	80.6	-5.2	(-12.3, 1.4)	4.7	2.6	0.575*	(0.505, 0.675)	1.777	7.3	2.6	0.789*	(0.654, 0.813)
Iringa	92.9	94.9	93.0	1.9	(-2.4, 6.2)	0.6	1.0	-0.447*	(-0.552, -0.292)	0.640	1.0	1.0	-0.178	(-0.217, 0.094)
Kagera	80.4	80.2	80.1	0	(-7.9, 11.8)	1.4	4.2	-0.894*	(-1.249, -0.939)	0.409	1.4	4.2	-1.127*	(-1.199, -1.064)
Katavi	76.6	71.0	76.2	-5.2	(-10.9, 1.8)	6.7	6.4	0.051	(-0.03, 0.112)	1.052	9.8	6.4	0.237*	(0.188, 0.314)
Kigoma	93.1	91.4	93.1	-1.7	(-5.2, 2.2)	0.8	0.9	-0.034	(-0.151, 0.05)	0.967	1.1	0.9	0.132	(-0.134, 0.152)
Kilimanjaro	87.7	91.1	87.5	3.7	(0, 11.5)	0.8	2.6	-1.091*	(-1.27, -1.014)	0.336	2.0	2.6	-0.633*	(-0.85, -0.532)
Lindi	76.9	73.0	76.8	-3.8	(-9.7, 1.8)	8.8	5.9	0.392*	(0.35, 0.446)	1.480	10.3	5.9	0.487*	(0.452, 0.538)
Manyara	98.7	96.6	98.7	-2.1*	(-4.2, -0.5)	0.2	0.1	1.561*	(1.415, 1.59)	4.761	0.7	0.1	2.156*	(2.038, 2.43)
Mara	65.7	68.1	65.7	2.4	(-3.9, 8.5)	2.5	2.2	-0.0003*	(0.041, 0.264)	1.000	3.1	2.2	0.242*	(0.167, 0.341)
Mbeya	79.1	81.3	79.1	2.2	(-7.7, 14)	1.6	5.7	-1.165*	(-1.41, -1.11)	0.312	2.1	5.7	-1.055*	(-1.103, -0.943)
Morogoro	83.2	86.1	83.6	2.4	(-2.8, 7.9)	1.6	2.6	-0.504*	(-0.599, -0.434)	0.604	2.4	2.7	-0.351*	(-0.43, -0.351)
Mtwara	88.9	82.1	88.8	-6.6*	(-12.3, -2.4)	2.5	1.2	0.796*	(0.627, 0.838)	2.217	7.0	1.2	1.411*	(1.196, 1.445)
Mwanza	91.9	93.3	92.3	1	(-3.8, 7.1)	0.4	1.4	-1.175*	(-1.305, -1.024)	0.309	0.6	1.4	-0.969*	(-1.15, -0.932)
Njombe	90.6	91.9	90.3	1.6	(-3.5, 7.7)	0.9	1.9	-0.661*	(-0.828, -0.591)	0.516	1.1	1.9	-0.659*	(-0.846, -0.611)
Pwani	80.6	76.8	80.4	-3.6	(-10.4, 4.6)	3.1	4.9	-0.521*	(-0.546, -0.366)	0.594	4.5	4.9	-0.215	(-0.25, 0.009)
Rukwa	79.5	82.8	79.6	3.2	(-3.9, 11.1)	2.5	3.6	-0.389*	(-0.453, -0.238)	0.678	3.6	3.6	-0.23*	(-0.286, -0.104)
Ruvuma	96.2	94.5	96.2	-1.7	(-3.8, 0.5)	0.2	0.2	0.053	(-0.031, 0.188)	1.055	0.5	0.2	0.519*	(0.4, 0.638)
Shinyanga	74.7	70.9	74.7	-3.8	(-11.9, 3.9)	3.4	2.2	0.382*	(0.283, 0.526)	1.465	4.8	2.2	0.537*	(0.437, 0.611)
Simiyu	86.1	87.9	86.3	1.6	(-5.5, 10)	1.9	3.8	-0.677*	(-0.798, -0.57)	0.508	2.2	3.8	-0.531*	(-0.616, -0.497)
Singida	89.3	92.8	89.3	3.5	(-3.1, 14.5)	1.4	6.1	-1.453*	(-1.609, -1.395)	0.234	2.5	6.1	-1.129*	(-1.164, -0.889)
Tabora	82.3	82.4	82.4	0	(-4.5, 5.1)	1.3	2.2	-0.557*	(-0.648, -0.466)	0.573	1.3	2.2	-0.576*	(-0.598, -0.481)
Tanga	86.9	87.3	86.8	0.5	(-5.3, 6.4)	1.5	2.1	-0.348*	(-0.461, -0.255)	0.706	1.5	2.1	-0.329*	(-0.532, -0.286)
National	85.3	85.7	85.4	0.3	(-1.2, 1.9)	0.1	0.1	-0.453*	(-0.564, -0.314)	0.636	0.1	0.1	-0.326*	(-0.532, -0.321)

Note: * indicates p-value <0.05, MSE = mean squared error, CI = confidence interval

Supplementary Table 7: Comparison of bias, variance, and MSE for weighted vs. unweighted estimates for the indicator "Provider performed assessment of client weight during the ANC consultation", by region

				BIAS		VARIANCE					MEAN SQUARED ERROR (MSE)			
Column 1	2	3	4	5	6	7	8	9	10	11	12	13	14	15

Region	Weighted TRUTH (x 10^2)	Average Unweighted Simulation Estimate (x 10^2)	Average Weighted Simulation Estimate (x 10^2)	Mean Difference in Bias (x 10^2)	95% CI Mean Difference in Bias	Variance of Unweighted Estimate (x 10^3)	Variance of Weighted Estimate (x 10^3	Log of the Ratio of the Variances (Unweighted/ Weighted)	95% CI of Log of the Ratio of the Variances	Ratio of the Variances (Unweighted/ Weighted)	MSE of Unweighted Estimates (x 10^3)	MSE of Weighted Estimates (x 10^3)	Log of the Ratio of the MSEs (Unweighted/ Weighted)	95% CI of Log of the Ratio of the MSEs
Arusha	78.0	91.8	80.2	11.6	(-0.5, 31)	3.4	22.9	-1.885*	(-1.991, -1.84)	0.152	22.4	23.3	-0.529*	(-0.575, -0.411)
Dar es Salaam	98.6	92.6	98.2	-5.6*	(-11.4, 0)	1.5	0.2	2.232*	(1.923, 2.274)	9.321	5.0	0.2	2.914*	(2.848, 3.142)
Dodoma	92.9	93.4	92.9	0.5	(-5, 5.4)	1.1	1.7	-0.35*	(-0.552, -0.301)	0.704	1.1	1.7	-0.425*	(-0.458, -0.219)
Geita	69.5	75.6	71.9	3.6	(-8.7, 15.4)	11.1	20.2	-0.59*	(-0.662, -0.537)	0.554	14.8	20.8	-0.48*	(-0.478, -0.383)
Iringa	96.9	92.7	96.8	-4.1*	(-11.7, 0)	3.0	0.6	1.667*	(1.454, 1.682)	5.296	4.8	0.6	1.78*	(1.711, 1.842)
Kagera	73.2	82.7	73.6	9.1	(-6.8, 26.7)	4.3	16.9	-1.289*	(-1.485, -1.268)	0.276	13.2	16.9	-0.681*	(-1.038, -0.59)
Katavi	79.6	85.6	79.2	6.3*	(0.9, 16.1)	5.9	11.8	-0.725*	(-0.747, -0.641)	0.485	9.5	11.8	-0.411*	(-0.426, -0.245)
Kigoma	85.5	82.1	85.2	-3.1	(-10.9, 5.4)	3.2	3.1	-0.002	(-0.1, 0.176)	0.998	4.4	3.1	0.202	(-0.069, 0.286)
Kilimanjaro	86.5	95.6	87.1	8.5*	(0, 25.6)	1.1	10.4	-2.309*	(-2.344, -2.158)	0.099	9.2	10.4	-0.684*	(-0.889, -0.628)
Lindi	75.6	81.1	75.9	5.1	(-1.8, 13.4)	6.2	8.5	-0.239*	(-0.395, -0.243)	0.787	9.2	8.5	-0.081*	(-0.154, -0.025)
Manyara	88.6	85.8	88.7	-2.9	(-9.9, 4.2)	2.1	2.2	0.112	(-0.157, 0.112)	1.118	2.9	2.2	0.109*	(0.009, 0.216)
Mara	85.1	80.5	85.0	-4.5	(-10.9, 0.7)	9.1	7.5	0.225*	(0.138, 0.244)	1.253	11.2	7.5	0.299*	(0.206, 0.303)
Mbeya	45.5	76.6	46.5	30.1*	(7.3, 48.9)	8.6	32.3	-1.362*	(-1.428, -1.226)	0.256	105.8	32.4	0.597*	(0.557, 0.685)
Morogoro	88.2	87.1	87.9	-0.8	(-8.6, 9.1)	2.9	4.0	-0.299*	(-0.453, -0.164)	0.741	3.1	4.0	-0.293*	(-0.369, -0.194)
Mtwara	84.4	87.1	85.0	2	(-2.9, 8.2)	5.8	7.7	-0.279*	(-0.338, -0.225)	0.756	6.6	7.8	-0.179*	(-0.205, -0.164)
Mwanza	73.8	87.9	74.7	13.1	(-4.7, 34.8)	4.1	23.5	-1.677*	(-1.858, -1.636)	0.187	23.9	23.6	-0.549*	(-0.632, -0.316)
Njombe	86.1	84.8	85.8	-1	(-9.6, 9.1)	4.5	5.3	-0.181*	(-0.28, -0.082)	0.834	4.6	5.4	-0.154*	(-0.305, -0.14)
Pwani	100.0	100.0	100.0	N/A	N/A	N/A	N/A	N/A	N/A	N/A	N/A	N/A	N/A	N/A
Rukwa	66.1	82.6	67.2	15.4*	(0.1, 31)	10.1	27.5	-0.968*	(-1.074, -0.905)	0.380	37.2	27.6	-0.172*	(-0.248, -0.045)
Ruvuma	96.8	98.0	96.7	1.3	(-1.6, 6)	0.2	0.8	-1.509*	(-1.582, -1.305)	0.221	0.3	0.8	-1.124*	(-1.229, -1.118)
Shinyanga	79.9	84.9	79.8	5.1	(-7.3, 21.5)	5.3	16.1	-1.053*	(-1.211, -1.042)	0.349	7.7	16.1	-0.936*	(-0.983, -0.707)
Simiyu	59.2	71.9	60.3	11.6	(-5.4, 27.6)	7.4	18.3	-0.938*	(-1.028, -0.791)	0.392	23.7	18.5	-0.143*	(-0.184, -0.047)
Singida	73.5	85.2	77.1	8.1	(-5, 26.3)	8.1	29.3	-1.212*	(-1.345, -1.219)	0.298	21.7	30.6	-0.721*	(-0.772, -0.7)
Tabora	82.0	87.5	82.7	4.8	(-1, 13.4)	3.4	7.6	-0.821*	(-0.872, -0.735)	0.440	6.4	7.7	-0.498*	(-0.494, -0.208)
Tanga	92.9	90.6	92.9	-2.3	(-6.7, 0.8)	2.1	1.6	0.272*	(0.197, 0.381)	1.313	2.6	1.6	0.402*	(0.402, 0.702)
National	80.8	86.0	80.8	5.2*	(1.8, 8.7)	0.2	0.7	-1.172*	(-1.36, -1.134)	0.310	2.9	0.7	0.811*	(0.734, 0.951)

Note: * indicates p-value <0.05, MSE = mean squared error, CI = confidence interval

Supplementary Table 8: Comparison of bias, variance, and MSE for weighted vs. unweighted estimates for the indicator "Provider performed RPR (syphilis test) laboratory investigation", by region

				BIAS		VARIANCE					MEAN SQUARED ERROR (MSE)			
Column 1	2	3	4	5	6	7	8	9	10	11	12	13	14	15

Region	Weighted TRUTH (x 10^2)	Average Unweighted Simulation Estimate (x 10^2)	Average Weighted Simulation Estimate (x 10^2)	Mean Difference in Bias (x 10^2)	95% CI Mean Difference in Bias	Variance of Unweighted Estimate (x 10^3)	Variance of Weighted Estimate (x 10^3	Log of the Ratio of the Variances (Unweighted/ Weighted)	95% CI of Log of the Ratio of the Variances	Ratio of the Variances (Unweighted/ Weighted)	MSE of Unweighted Estimates (x 10^3)	MSE of Weighted Estimates (x 10^3)	Log of the Ratio of the MSEs (Unweighted/ Weighted)	95% CI of Log of the Ratio of the MSEs
Arusha	11.1	9.7	11.4	-1.6	(-12.3, 7.5)	3.3	4.5	-0.307*	(-0.49, -0.155)	0.735	3.4	4.5	-0.251*	(-0.395, -0.202)
Dar es Salaam	1.7	8.4	2.0	6.4*	(0, 13.5)	2.1	0.3	2.258*	(1.853, 2.396)	9.566	6.5	0.3	2.802*	(2.708, 3.022)
Dodoma	21.0	27.3	21.3	6*	(0.5, 13.3)	8.8	7.2	0.201*	(0.144, 0.281)	1.223	12.8	7.2	0.484*	(0.329, 0.545)
Geita	0.0	0.0	0.0	N/A	N/A	N/A	N/A	N/A	N/A	N/A	N/A	N/A	N/A	N/A
Iringa	6.9	14.8	7.3	7.5*	(0, 17.3)	6.7	2.2	1.169*	(1.035, 1.217)	3.218	12.9	2.2	1.535*	(1.41, 1.697)
Kagera	9.4	8.0	9.5	-1.5	(-10, 6.4)	2.9	2.9	0.101	(-0.139, 0.14)	1.106	3.1	2.9	-0.012	(-0.161, 0.037)
Katavi	15.7	18.1	17.0	1.1	(-5.8, 9)	7.6	7.1	0.103*	(0.003, 0.151)	1.109	8.2	7.2	0.126*	(0.083, 0.201)
Kigoma	9.2	16.0	9.4	6.6	(-0.6, 14.1)	3.8	2.4	0.48*	(0.348, 0.627)	1.617	8.5	2.4	1.008*	(0.913, 1.077)
Kilimanjaro	56.6	50.2	54.7	-4.5	(-15.6, 0.2)	17.3	15.6	0.038*	(0.047, 0.17)	1.039	21.4	16.0	0.182*	(0.147, 0.2)
Lindi	36.5	39.6	35.8	3.8	(-5.6, 12.2)	11.6	10.4	0.161*	(0.048, 0.184)	1.175	12.5	10.4	0.187*	(0.185, 0.286)
Manyara	21.4	16.6	19.2	-2.7	(-20.4, 9.9)	5.8	19.0	-1.217*	(-1.274, -1.098)	0.296	8.2	19.5	-1.043*	(-1.126, -0.898)
Mara	3.5	1.5	3.7	-2.3*	(-8, 0)	0.2	1.4	-1.81*	(-1.903, -1.768)	0.164	0.6	1.4	-1.211*	(-1.351, -1.057)
Mbeya	1.9	9.8	2.2	7.6*	(0, 16.2)	3.0	0.3	2.352*	(2.115, 2.507)	10.505	9.3	0.3	3.047*	(2.64, 3.053)
Morogoro	24.5	19.7	24.4	-4.7	(-20.7, 8)	7.9	18.8	-0.944*	(-0.968, -0.768)	0.389	10.2	18.8	-0.762*	(-0.825, -0.718)
Mtwara	55.8	61.1	55.0	6.1	(-3.4, 15.6)	11.6	13.6	-0.132*	(-0.234, -0.084)	0.877	14.5	13.6	-0.028	(-0.04, 0.121)
Mwanza	0.6	3.1	0.7	2.4*	(0, 7.3)	0.8	0.0	2.894*	(2.711, 3.003)	18.070	1.4	0.0	3.176*	(3.142, 3.245)
Njombe	67.8	61.8	67.0	-5.2	(-20.3, 13.1)	17.3	19.3	-0.126*	(-0.214, -0.006)	0.882	20.8	19.3	-0.013	(-0.056, 0.131)
Pwani	26.4	32.0	26.7	5.3	(-11.7, 17.5)	18.7	17.2	0.156	(-0.003, 0.166)	1.169	21.8	17.2	0.179*	(0.047, 0.239)
Rukwa	0.7	1.9	0.8	1.1*	(0, 3.8)	0.4	0.1	1.717*	(1.589, 1.795)	5.568	0.5	0.1	1.887*	(1.809, 1.96)
Ruvuma	22.0	17.7	21.2	-3.5	(-16.6, 6.6)	3.1	9.1	-1.146*	(-1.198, -0.977)	0.318	4.9	9.2	-0.822*	(-0.968, -0.749)
Shinyanga	7.1	12.3	8.0	4.3	(-5.1, 13.2)	3.5	2.7	0.292*	(0.08, 0.46)	1.339	6.2	2.8	0.6*	(0.514, 0.772)
Simiyu	12.1	15.9	12.3	3.6	(-9.4, 18.1)	6.0	7.7	-0.137*	(-0.386, -0.118)	0.872	7.4	7.7	-0.21*	(-0.317, -0.161)
Singida	51.9	42.3	50.5	-8.2	(-22.6, 5.2)	11.9	13.8	-0.196*	(-0.247, -0.049)	0.822	21.0	14.0	0.181	(-0.022, 0.212)
Tabora	2.3	5.7	2.4	3.3*	(0, 9.4)	1.7	0.4	1.356*	(1.316, 1.599)	3.882	2.9	0.4	1.653*	(1.491, 1.652)
Tanga	23.2	29.7	23.2	6.5	(-6.9, 18.5)	12.8	11.3	0.06*	(0.029, 0.235)	1.062	17.0	11.3	0.28*	(0.276, 0.384)
National	13.7	17.4	13.6	3.8*	(1.4, 6.2)	0.2	0.3	-0.129	(-0.192, 0.042)	0.879	1.6	0.3	1.268*	(1.255, 1.536)

Note: * indicates p-value <0.05, MSE = mean squared error, CI = confidence interval

Supplementary Table 9: Comparison of bias, variance, and MSE for weighted vs. unweighted estimates for the indicator "Provider prescribed or gave tetanus toxoid injection", by region

				**BIAS**		**VARIANCE**					**MEAN SQUARED ERROR (MSE)**			
Column 1	2	3	4	5	6	7	8	9	10	11	12	13	14	15

Region	Weighted TRUTH (x 10^2)	Average Unweighted Simulation Estimate (x 10^2)	Average Weighted Simulation Estimate (x 10^2)	Mean Difference in Bias (x 10^2)	95% CI Mean Difference in Bias	Variance of Unweighted Estimate (x 10^3)	Variance of Weighted Estimate (x 10^3	Log of the Ratio of the Variances (Unweighted/ Weighted)	95% CI of Log of the Ratio of the Variances	Ratio of the Variances (Unweighted/ Weighted)	MSE of Unweighted Estimates (x 10^3)	MSE of Weighted Estimates (x 10^3)	Log of the Ratio of the MSEs (Unweighted/ Weighted)	95% CI of Log of the Ratio of the MSEs
Arusha	60.1	66.8	61.3	5.5	(-6.2, 16.7)	5.2	8.0	-0.371*	(-0.537, -0.304)	0.690	9.7	8.1	-0.062*	(-0.217, -0.034)
Dar es Salaam	77.3	58.3	76.0	-17.7	(-33.5, 6.2)	6.2	17.2	-0.956*	(-1.178, -0.827)	0.384	42.3	17.3	0.384	(-0.086, 0.449)
Dodoma	72.5	74.0	73.0	1	(-7.6, 9.5)	4.9	5.9	-0.149*	(-0.282, -0.081)	0.861	5.1	5.9	-0.152*	(-0.162, -0.041)
Geita	53.7	55.0	53.8	1.1	(-8, 9.4)	7.4	6.5	0.181*	(0.031, 0.231)	1.198	7.6	6.5	0.114*	(0.068, 0.152)
Iringa	51.0	51.4	50.9	0.5	(-16.5, 16)	7.6	13.6	-0.71*	(-0.712, -0.457)	0.492	7.6	13.6	-0.578*	(-0.727, -0.49)
Kagera	62.3	58.3	61.0	-2.7	(-16.2, 13.4)	5.7	11.0	-0.582*	(-0.781, -0.529)	0.559	7.2	11.1	-0.507*	(-0.53, -0.316)
Katavi	69.9	65.5	69.2	-3.7	(-9.9, 3)	7.2	6.8	0.001	(-0.004, 0.12)	1.001	9.2	6.8	0.174*	(0.157, 0.254)
Kigoma	26.6	33.6	26.7	6.9	(-2, 14.6)	3.5	5.3	-0.478*	(-0.514, -0.306)	0.620	8.5	5.3	0.137	(-0.102, 0.171)
Kilimanjaro	59.1	64.2	58.6	5.6	(-0.2, 18.4)	8.8	11.7	-0.332*	(-0.362, -0.202)	0.717	11.5	11.8	-0.107*	(-0.174, -0.059)
Lindi	73.5	77.9	73.8	4.1	(-3.3, 11.2)	5.5	7.9	-0.326*	(-0.457, -0.3)	0.722	7.4	8.0	-0.193*	(-0.285, -0.062)
Manyara	69.1	66.0	69.0	-3	(-11.5, 5.7)	2.3	3.8	-0.471*	(-0.641, -0.411)	0.625	3.2	3.8	-0.337*	(-0.352, -0.17)
Mara	50.7	59.3	50.9	8.4	(-1.6, 17.2)	4.5	8.4	-0.657*	(-0.713, -0.516)	0.518	12.0	8.4	0.006	(-0.215, 0.059)
Mbeya	47.7	66.3	49.2	17.1	(-6.7, 35.2)	8.9	27.3	-1.08*	(-1.239, -1.027)	0.339	43.6	27.5	-0.04	(-0.078, 0.135)
Morogoro	56.1	63.5	56.9	6.6	(-5.6, 19.9)	6.9	13.0	-0.569*	(-0.732, -0.54)	0.566	12.3	13.1	-0.359*	(-0.41, -0.226)
Mtwara	56.7	59.5	56.7	2.8	(-5.7, 10.4)	6.1	8.4	-0.331*	(-0.412, -0.245)	0.718	6.9	8.4	-0.296*	(-0.341, -0.234)
Mwanza	56.0	55.0	56.2	-1.2	(-17.3, 16.9)	6.1	13.2	-0.838*	(-0.902, -0.65)	0.432	6.2	13.2	-0.748*	(-0.79, -0.647)
Njombe	27.5	43.4	29.3	14.1*	(1.2, 29.3)	15.2	11.1	0.289*	(0.204, 0.418)	1.335	40.6	11.5	0.883*	(0.769, 0.924)
Pwani	20.8	26.1	21.7	4.4	(-7.8, 14.9)	9.5	12.2	-0.251*	(-0.365, -0.145)	0.778	12.3	12.3	-0.142*	(-0.321, -0.135)
Rukwa	82.7	81.2	81.7	-0.5	(-9.6, 11.6)	3.1	6.0	-0.733*	(-0.793, -0.557)	0.481	3.3	6.1	-0.655*	(-0.738, -0.631)
Ruvuma	37.8	48.8	38.7	10.1	(-1.7, 21.1)	3.2	7.1	-0.807*	(-0.917, -0.689)	0.446	15.2	7.2	0.217*	(0.119, 0.239)
Shinyanga	65.9	64.2	64.8	-0.7	(-12.3, 12)	3.9	10.3	-1.009*	(-1.08, -0.866)	0.365	4.2	10.4	-0.947*	(-0.963, -0.798)
Simiyu	35.8	57.5	36.8	20.7*	(4.3, 37.3)	8.4	11.8	-0.382*	(-0.475, -0.2)	0.683	55.5	11.9	1.026*	(0.984, 1.161)
Singida	48.0	46.7	47.7	-1	(-10, 6.7)	4.4	5.7	-0.242*	(-0.386, -0.136)	0.785	4.6	5.7	-0.252*	(-0.504, -0.247)
Tabora	56.3	64.2	56.3	8*	(0.2, 16.8)	4.5	8.2	-0.625*	(-0.678, -0.512)	0.536	10.8	8.2	-0.025	(-0.13, 0.008)
Tanga	57.4	57.0	56.8	0.1	(-16.2, 16.3)	7.0	16.9	-0.858*	(-0.996, -0.751)	0.424	7.0	16.9	-0.879*	(-1.018, -0.857)
National	53.5	58.1	53.6	4.5*	(1.1, 8)	0.3	0.6	-0.922*	(-0.937, -0.671)	0.398	2.4	0.6	0.746*	(0.598, 0.772)

Note: * indicates p-value <0.05, MSE = mean squared error, CI = confidence interval

Supplementary Table 10: Comparison of bias, variance, and MSE for weighted vs. unweighted estimates for the indicator "Facility has capacity to conduct hemoglobin testing on-site", by region

				**BIAS**		**VARIANCE**					**MEAN SQUARED ERROR (MSE)**			
Column 1	2	3	4	5	6	7	8	9	10	11	12	13	14	15

Region	Weighted TRUTH (x 10^2)	Average Unweighted Simulation Estimate (x 10^2)	Average Weighted Simulation Estimate (x 10^2)	Mean Difference in Bias (x 10^2)	95% CI Mean Difference in Bias	Variance of Unweighted Estimate (x 10^3)	Variance of Weighted Estimate (x 10^3	Log of the Ratio of the Variances (Unweighted/ Weighted)	95% CI of Log of the Ratio of the Variances	Ratio of the Variances (Unweighted/ Weighted)	MSE of Unweighted Estimates (x 10^3)	MSE of Weighted Estimates (x 10^3)	Log of the Ratio of the MSEs (Unweighted/ Weighted)	95% CI of Log of the Ratio of the MSEs
Arusha	29.2	56.4	29.6	26.9*	(9.7, 40.9)	6.7	11.4	-0.383*	(-0.669, -0.402)	0.682	80.6	11.5	1.301*	(1.144, 1.316)
Dar es Salaam	89.0	91.7	89.1	2.6	(-6, 16.3)	1.6	5.9	-1.467*	(-1.465, -1.158)	0.231	2.3	5.9	-1.103*	(-1.175, -0.96)
Dodoma	42.2	53.0	41.9	11.2	(-0.6, 23)	6.1	10.1	-0.482*	(-0.609, -0.403)	0.618	17.8	10.1	0.186	(-0.176, 0.235)
Geita	19.4	27.0	19.3	7.7	(-4.3, 21.4)	6.4	8.3	-0.302*	(-0.364, -0.127)	0.740	12.2	8.3	0.127*	(0.106, 0.296)
Iringa	23.6	55.5	23.9	31.6*	(20.8, 42.7)	7.3	7.2	0.068	(-0.088, 0.119)	1.070	109.0	7.2	2.132*	(1.871, 2.208)
Kagera	19.1	53.4	19.2	34.2*	(22.2, 44.7)	5.8	4.8	0.218*	(0.029, 0.31)	1.243	123.6	4.9	2.551*	(2.481, 2.737)
Katavi	26.0	31.1	25.9	5.2	(-0.8, 10.6)	6.6	6.2	0.033*	(0.003, 0.141)	1.034	9.3	6.2	0.225*	(0.128, 0.254)
Kigoma	9.4	39.9	9.7	30.2*	(20, 39.5)	5.3	0.9	1.796*	(1.719, 1.925)	6.028	98.6	0.9	4.085*	(3.947, 4.106)
Kilimanjaro	16.6	46.6	16.8	29.9*	(18, 41.8)	5.8	4.3	0.246*	(0.162, 0.433)	1.279	96.1	4.3	2.467*	(2.432, 2.637)
Lindi	30.1	53.9	30.4	23.5*	(10.4, 36.7)	6.7	8.8	-0.192*	(-0.397, -0.173)	0.825	63.7	8.8	1.381*	(1.211, 1.401)
Manyara	35.2	49.7	35.2	14.5	(-3.1, 32.3)	7.4	15.5	-0.689*	(-0.872, -0.622)	0.502	28.3	15.5	0.098	(-0.117, 0.102)
Mara	23.6	58.2	24.6	33.5*	(20.3, 43.3)	6.9	7.3	-0.004	(-0.19, 0.061)	0.996	126.3	7.4	2.204*	(2.069, 2.29)
Mbeya	16.3	54.6	16.5	38.1*	(27.5, 47.8)	4.9	3.6	0.255*	(0.158, 0.446)	1.291	151.3	3.6	3.031*	(3.004, 3.115)
Morogoro	30.4	58.7	31.0	27.8*	(13.5, 39.5)	5.4	8.9	-0.528*	(-0.629, -0.361)	0.590	85.9	8.9	1.577*	(1.573, 1.743)
Mtwara	13.2	41.5	13.8	27.8*	(15.9, 39.1)	7.7	3.2	0.963*	(0.75, 1.025)	2.619	88.0	3.2	2.701*	(2.683, 2.826)
Mwanza	28.2	58.3	29.0	29.3*	(12.4, 44.7)	7.3	12.1	-0.536*	(-0.65, -0.359)	0.585	97.6	12.1	1.417*	(1.405, 1.525)
Njombe	9.9	43.4	10.5	32.9*	(22.6, 42.4)	6.9	1.3	1.72*	(1.596, 1.79)	5.585	119.4	1.3	3.908*	(3.844, 4.027)
Pwani	35.6	64.2	35.3	28.9*	(17.9, 41.2)	6.3	10.2	-0.426*	(-0.594, -0.383)	0.653	87.7	10.2	1.561*	(1.561, 1.71)
Rukwa	27.3	44.0	28.0	16.1*	(4.3, 26.8)	7.4	8.1	-0.083	(-0.191, 0.006)	0.920	35.2	8.2	0.916*	(0.879, 1.323)
Ruvuma	27.7	43.0	27.5	15.5*	(3.3, 27.6)	6.0	8.3	-0.196*	(-0.44, -0.19)	0.822	29.6	8.3	0.774*	(0.691, 0.839)
Shinyanga	30.6	53.2	30.5	22.7*	(8.6, 36.8)	9.4	11.4	-0.184*	(-0.32, -0.075)	0.832	60.7	11.4	1.107*	(1.034, 1.198)
Simiyu	24.4	31.9	23.8	8.1	(-2.1, 20.4)	6.9	7.8	-0.078*	(-0.224, -0.013)	0.925	12.7	7.8	0.187	(-0.04, 0.202)
Singida	15.3	40.5	16.1	24.4*	(10.4, 37.1)	7.8	5.8	0.206*	(0.154, 0.423)	1.228	71.4	5.9	1.909*	(1.838, 1.962)
Tabora	58.3	63.7	58.0	5.7	(-4.3, 16.9)	5.8	9.5	-0.465*	(-0.588, -0.389)	0.628	8.7	9.6	-0.232*	(-0.348, -0.141)
Tanga	29.1	58.2	29.0	29.3*	(17.1, 41.6)	5.5	7.6	-0.36*	(-0.427, -0.171)	0.698	90.2	7.6	1.919*	(1.739, 1.92)
National	29.5	52.0	29.4	22.6*	(19.7, 25.5)	0.3	0.4	-0.269*	(-0.493, -0.224)	0.764	51.1	0.4	4.258*	(4.256, 4.515)

Note: * indicates p-value <0.05, MSE = mean squared error, CI = confidence interval

Supplementary Table 11: Comparison of bias, variance, and MSE for weighted vs. unweighted estimates for the indicator "Facility has TT vaccine in-stock", by region

				BIAS		VARIANCE					MEAN SQUARED ERROR (MSE)			
Column 1	2	3	4	5	6	7	8	9	10	11	12	13	14	15

Region	Weighted TRUTH (x 10^2)	Average Unweighted Simulation Estimate (x 10^2)	Average Weighted Simulation Estimate (x 10^2)	Mean Difference in Bias (x 10^2)	95% CI Mean Difference in Bias	Variance of Unweighted Estimate (x 10^3)	Variance of Weighted Estimate (x 10^3	Log of the Ratio of the Variances (Unweighted/ Weighted)	95% CI of Log of the Ratio of the Variances	Ratio of the Variances (Unweighted/ Weighted)	MSE of Unweighted Estimates (x 10^3)	MSE of Weighted Estimates (x 10^3)	Log of the Ratio of the MSEs (Unweighted/ Weighted)	95% CI of Log of the Ratio of the MSEs
Arusha	91.2	94.6	90.8	3.8	(-3.5, 15.4)	1.4	5.9	-1.432*	(-1.545, -1.341)	0.239	2.5	5.9	-1.09*	(-1.162, -1.025)
Dar es Salaam	89.0	89.9	89.4	0.5	(-8.9, 13.5)	1.8	5.6	-1.115*	(-1.275, -0.967)	0.328	1.9	5.6	-1.089*	(-1.165, -1.066)
Dodoma	90.4	91.3	90.5	0.8	(-6.2, 9.1)	1.7	3.4	-0.626*	(-0.82, -0.563)	0.535	1.8	3.4	-0.636*	(-0.652, -0.455)
Geita	90.1	86.6	89.9	-3.3	(-13, 7.3)	3.8	5.1	-0.341*	(-0.445, -0.151)	0.711	5.1	5.1	-0.113*	(-0.354, -0.111)
Iringa	88.6	94.7	89.1	5.5*	(0, 15.3)	1.4	5.4	-1.375*	(-1.444, -1.321)	0.253	5.0	5.4	-0.619*	(-0.728, -0.424)
Kagera	99.4	97.6	99.4	-1.8	(-5.8, 0)	0.5	0.0	2.736*	(2.643, 2.769)	15.425	0.8	0.0	2.964*	(2.939, 3.035)
Katavi	94.3	94.0	94.6	-0.6	(-3.9, 2.1)	1.6	1.5	0.05	(-0.038, 0.121)	1.051	1.6	1.5	0.077*	(0.073, 0.187)
Kigoma	93.1	90.0	93.1	-3.2	(-10.7, 3.4)	2.3	2.2	0.031	(-0.08, 0.153)	1.032	3.3	2.2	0.258*	(0.069, 0.462)
Kilimanjaro	91.6	90.9	91.8	-0.9	(-9.2, 8.6)	2.2	3.7	-0.606*	(-0.697, -0.414)	0.546	2.2	3.7	-0.522*	(-0.562, -0.46)
Lindi	72.8	81.1	73.1	8	(-2.1, 19.6)	4.1	9.1	-0.728*	(-0.887, -0.676)	0.483	11.1	9.1	-0.146*	(-0.333, -0.128)
Manyara	90.7	91.0	91.3	-0.3	(-9.7, 11.9)	2.6	5.4	-0.8*	(-0.915, -0.584)	0.449	2.6	5.4	-0.703*	(-0.709, -0.378)
Mara	90.0	89.5	90.1	-0.7	(-9, 12.4)	2.8	5.8	-0.606*	(-0.866, -0.563)	0.546	2.8	5.8	-0.724*	(-0.725, -0.473)
Mbeya	76.4	88.2	76.9	11.3*	(0.7, 23.9)	1.8	8.1	-1.456*	(-1.623, -1.413)	0.233	15.6	8.1	0.065*	(0.032, 0.204)
Morogoro	65.4	80.8	66.5	14.3*	(1.2, 29.1)	3.4	11.4	-1.276*	(-1.307, -1.103)	0.279	27.1	11.6	0.312*	(0.28, 0.584)
Mtwara	85.1	91.0	84.8	6.2*	(0, 13.1)	2.5	6.3	-0.954*	(-1, -0.866)	0.385	6.0	6.3	-0.362*	(-0.495, -0.225)
Mwanza	98.5	94.4	98.5	-4*	(-9.4, 0)	1.4	0.2	2.113*	(2.101, 2.315)	8.271	3.1	0.2	2.731*	(2.619, 2.733)
Njombe	94.3	97.1	93.9	3.2	(0, 9.9)	0.7	3.1	-1.474*	(-1.541, -1.428)	0.229	1.5	3.1	-1.042*	(-1.287, -1.042)
Pwani	93.8	94.0	93.7	0.4	(-7.4, 7)	1.5	2.9	-0.526*	(-0.732, -0.496)	0.591	1.6	2.9	-0.587*	(-0.622, -0.439)
Rukwa	84.5	88.1	84.3	3.8	(-3.5, 11.7)	2.9	5.7	-0.624*	(-0.758, -0.589)	0.536	4.2	5.7	-0.512*	(-0.665, -0.472)
Ruvuma	78.3	85.0	78.1	6.9	(-2.4, 17.4)	2.9	7.0	-0.891*	(-0.987, -0.768)	0.410	7.4	7.0	-0.36*	(-0.573, -0.281)
Shinyanga	93.3	96.5	93.5	3*	(0, 10.5)	1.2	4.1	-1.256*	(-1.303, -1.187)	0.285	2.2	4.1	-0.932*	(-0.954, -0.69)
Simiyu	100.0	100.0	100.0	N/A	N/A	N/A	N/A	N/A	N/A	N/A	N/A	N/A	N/A	N/A
Singida	100.0	100.0	100.0	N/A	N/A	N/A	N/A	N/A	N/A	N/A	N/A	N/A	N/A	N/A
Tabora	69.2	78.4	69.3	9.1	(-0.3, 20)	4.5	9.3	-0.665*	(-0.826, -0.619)	0.514	12.9	9.3	-0.046*	(-0.2, -0.047)
Tanga	75.3	82.6	74.4	8.2	(-3.4, 21.7)	3.0	8.8	-1.159*	(-1.188, -0.944)	0.314	8.4	8.9	-0.466*	(-0.639, -0.418)
National	86.4	90.3	86.4	3.9*	(1.8, 5.9)	0.1	0.3	-1.109*	(-1.27, -1.029)	0.330	1.6	0.3	1.135*	(1.106, 1.236)

Note: * indicates p-value <0.05, MSE = mean squared error, CI = confidence interval

Supplementary Table 12: Comparison of bias, variance, and MSE for weighted vs. unweighted estimates for the indicator "Facility has IPT drug in-stock", by region

				BIAS		VARIANCE					MEAN SQUARED ERROR (MSE)			
Column 1	2	3	4	5	6	7	8	9	10	11	12	13	14	15

Region	Weighted TRUTH (x 10^2)	Average Unweighted Simulation Estimate (x 10^2)	Average Weighted Simulation Estimate (x 10^2)	Mean Difference in Bias (x 10^2)	95% CI Mean Difference in Bias	Variance of Unweighted Estimate (x 10^3)	Variance of Weighted Estimate (x 10^3	Log of the Ratio of the Variances (Unweighted/ Weighted)	95% CI of Log of the Ratio of the Variances	Ratio of the Variances (Unweighted/ Weighted)	MSE of Unweighted Estimates (x 10^3)	MSE of Weighted Estimates (x 10^3)	Log of the Ratio of the MSEs (Unweighted/ Weighted)	95% CI of Log of the Ratio of the MSEs
Arusha	51.1	56.5	51.5	4.9	(-12.9, 23.6)	5.9	13.8	-0.813*	(-0.983, -0.72)	0.444	8.8	13.8	-0.657*	(-0.82, -0.623)
Dar es Salaam	72.0	81.7	73.2	8.4	(-8.9, 27.9)	3.2	14.4	-1.541*	(-1.649, -1.381)	0.214	12.5	14.5	-0.634*	(-0.666, -0.583)
Dodoma	27.0	35.7	27.8	8	(-3.5, 19.6)	5.1	6.9	-0.277*	(-0.405, -0.174)	0.758	12.7	6.9	0.26*	(0.069, 0.256)
Geita	67.8	70.0	67.7	2.2	(-11.4, 15.9)	6.9	11.8	-0.514*	(-0.659, -0.422)	0.598	7.4	11.8	-0.444*	(-0.573, -0.377)
Iringa	99.5	97.2	99.5	-2.3	(-8.4, 0)	0.8	0.0	3.372*	(3.259, 3.419)	29.142	1.3	0.0	3.662*	(3.495, 3.663)
Kagera	91.1	87.8	91.2	-3.4	(-13, 6.2)	2.6	3.8	-0.521*	(-0.531, -0.26)	0.594	3.7	3.8	-0.222*	(-0.282, -0.119)
Katavi	79.7	74.9	79.7	-4.7	(-10.8, 0.8)	5.6	4.9	0.128*	(0.055, 0.214)	1.137	7.9	4.9	0.31*	(0.244, 0.365)
Kigoma	41.5	50.2	41.9	8.2	(-4.5, 19.9)	6.0	9.4	-0.376*	(-0.549, -0.346)	0.686	13.6	9.4	0.0390	(-0.128, 0.07)
Kilimanjaro	77.5	78.9	77.5	1.4	(-13.4, 16.2)	4.2	9.5	-0.883*	(-0.933, -0.684)	0.414	4.4	9.5	-0.788*	(-0.796, -0.663)
Lindi	70.3	70.0	70.7	-0.7	(-12.7, 11.6)	5.4	9.9	-0.564*	(-0.72, -0.493)	0.569	5.4	9.9	-0.58*	(-0.672, -0.55)
Manyara	50.4	53.7	51.0	2.7	(-14.9, 19.8)	7.6	17.0	-0.847*	(-0.908, -0.671)	0.429	8.7	17.0	-0.706*	(-0.892, -0.706)
Mara	62.5	63.2	62.5	0.7	(-17.2, 17.4)	5.7	14.1	-0.949*	(-1.042, -0.788)	0.387	5.7	14.1	-0.935*	(-0.957, -0.688)
Mbeya	68.8	76.2	69.0	7.2	(-7.6, 22.7)	3.7	10.4	-1.084*	(-1.165, -0.922)	0.338	9.1	10.4	-0.449*	(-0.498, -0.319)
Morogoro	75.7	84.3	75.1	9.2	(-4.2, 21.3)	2.4	8.7	-1.271*	(-1.404, -1.174)	0.281	9.8	8.8	-0.424*	(-0.432, -0.09)
Mtwara	69.4	79.2	68.8	10.4*	(0.4, 21.6)	5.1	11.0	-0.817*	(-0.839, -0.693)	0.442	14.7	11.0	-0.091*	(-0.405, -0.076)
Mwanza	67.4	55.6	67.8	-12.2	(-28.5, 6.4)	5.8	13.5	-0.841*	(-1.013, -0.691)	0.431	19.7	13.6	-0.0950	(-0.109, 0.172)
Njombe	80.8	83.9	80.9	3	(-7.5, 15.1)	3.6	8.2	-0.829*	(-0.942, -0.687)	0.437	4.6	8.2	-0.699*	(-0.775, -0.538)
Pwani	62.7	70.9	63.2	7.6	(-5.6, 20.8)	6.0	12.1	-0.661*	(-0.798, -0.607)	0.517	12.6	12.1	-0.235*	(-0.385, -0.137)
Rukwa	66.0	64.7	66.1	-1.4	(-13, 10.7)	6.8	10.4	-0.426*	(-0.521, -0.323)	0.653	7.0	10.4	-0.37*	(-0.387, -0.078)
Ruvuma	63.7	63.7	64.1	-0.4	(-13.2, 12.5)	6.2	10.6	-0.561*	(-0.628, -0.432)	0.571	6.2	10.6	-0.519*	(-0.576, -0.473)
Shinyanga	57.4	46.9	57.6	-10.7	(-24.6, 4.1)	10.1	15.7	-0.463*	(-0.547, -0.346)	0.629	21.1	15.7	-0.0380	(-0.032, 0.159)
Simiyu	72.6	74.1	72.6	1.6	(-8.9, 11.9)	6.0	8.6	-0.355*	(-0.458, -0.273)	0.701	6.2	8.6	-0.389*	(-0.39, -0.227)
Singida	47.5	65.7	48.0	17.7*	(1.7, 32.6)	7.3	16.7	-0.879*	(-0.918, -0.726)	0.415	40.4	16.7	0.376*	(0.319, 0.436)
Tabora	34.9	42.6	34.9	7.7	(-3.3, 19.5)	5.5	8.4	-0.402*	(-0.546, -0.322)	0.669	11.5	8.4	0.0550	(-0.045, 0.093)
Tanga	42.1	46.3	41.9	4.4	(-10.6, 18.3)	5.5	11.2	-0.565*	(-0.816, -0.583)	0.568	7.3	11.2	-0.61*	(-0.801, -0.565)
National	63.2	67.1	63.3	3.8*	(0.8, 7.1)	0.2	0.5	-0.854*	(-1.043, -0.799)	0.426	1.8	0.5	0.678*	(0.578, 0.743)

Note: * indicates p-value <0.05, MSE = mean squared error, CI = confidence interval

Supplementary Table 13: Comparison of bias, variance, and MSE for weighted vs. unweighted estimates for the indicator "Facility has ITNs or ITN vouchers in-stock", by region

				BIAS		VARIANCE					MEAN SQUARED ERROR (MSE)			
Column 1	2	3	4	5	6	7	8	9	10	11	12	13	14	15

Region	Weighted TRUTH (x 10^2)	Average Unweighted Simulation Estimate (x 10^2)	Average Weighted Simulation Estimate (x 10^2)	Mean Difference in Bias (x 10^2)	95% CI Mean Difference in Bias	Variance of Unweighted Estimate (x 10^3)	Variance of Weighted Estimate (x 10^3	Log of the Ratio of the Variances (Unweighted/ Weighted)	95% CI of Log of the Ratio of the Variances	Ratio of the Variances (Unweighted/ Weighted)	MSE of Unweighted Estimates (x 10^3)	MSE of Weighted Estimates (x 10^3)	Log of the Ratio of the MSEs (Unweighted/ Weighted)	95% CI of Log of the Ratio of the MSEs
Arusha	4.5	15.5	4.8	10.7*	(3.9, 18.7)	3.2	0.5	1.807*	(1.601, 1.909)	6.095	15.3	0.6	2.878*	(2.882, 3.038)
Dar es Salaam	3.6	14.1	3.6	10.6*	(3.5, 19.2)	2.5	0.3	2.161*	(1.98, 2.282)	8.682	13.7	0.3	3.36*	(3.315, 3.617)
Dodoma	1.6	6.8	1.6	5.1*	(0, 10.5)	1.4	0.1	2.657*	(2.514, 2.67)	14.259	4.1	0.1	3.327*	(3.308, 3.617)
Geita	2.0	6.8	2.1	4.7*	(0, 11.7)	2.0	0.2	2.092*	(2.126, 2.268)	8.103	4.3	0.2	2.652*	(2.488, 2.662)
Iringa	3.8	16.7	4.0	12.6*	(4.4, 21.1)	3.4	0.3	2.314*	(2.203, 2.425)	10.111	19.9	0.3	3.521*	(3.508, 3.798)
Kagera	18.7	26.8	18.9	7.9	(-5.3, 20.5)	4.8	7.3	-0.417*	(-0.556, -0.275)	0.659	11.4	7.3	0.107*	(0.08, 0.265)
Katavi	8.0	6.4	8.2	-1.8*	(-4.4, 0)	1.7	2.7	-0.434*	(-0.487, -0.425)	0.648	2.0	2.7	-0.396*	(-0.465, -0.383)
Kigoma	6.6	10.2	6.7	3.4	(-2.9, 11)	2.6	2.2	0.096*	(0.059, 0.305)	1.100	3.9	2.2	0.333*	(0.316, 0.578)
Kilimanjaro	30.3	29.9	29.4	0.5	(-14.6, 16.6)	4.4	10.6	-0.865*	(-1.023, -0.754)	0.421	4.4	10.7	-0.909*	(-1.075, -0.911)
Lindi	2.9	13.5	3.1	10.5*	(2.3, 18.7)	3.2	0.2	2.466*	(2.464, 2.655)	11.780	14.5	0.3	3.519*	(3.514, 3.654)
Manyara	7.6	25.3	8.3	17*	(8.2, 27.5)	5.9	1.4	1.568*	(1.339, 1.596)	4.798	37.2	1.4	2.666*	(2.55, 2.688)
Mara	2.5	7.7	2.5	5.1*	(0, 11.3)	1.8	0.3	1.922*	(1.789, 2.008)	6.835	4.5	0.3	2.41*	(2.204, 2.552)
Mbeya	7.6	13.4	7.5	5.9	(-5.6, 16)	2.4	3.0	-0.229*	(-0.38, -0.031)	0.795	5.8	3.0	0.402*	(0.07, 0.484)
Morogoro	14.7	17.7	14.9	2.9	(-7, 12.1)	3.4	5.5	-0.53*	(-0.606, -0.36)	0.589	4.3	5.5	-0.352*	(-0.457, -0.346)
Mtwara	3.6	17.5	3.7	13.8*	(4.5, 24.1)	4.7	0.3	2.768*	(2.625, 2.776)	15.931	24.1	0.3	3.846*	(3.841, 4.025)
Mwanza	19.1	38.7	19.9	18.8*	(5.5, 32.2)	6.8	7.9	-0.252*	(-0.303, -0.009)	0.777	45.0	7.9	1.211*	(1.195, 1.36)
Njombe	5.4	21.9	5.9	16.1*	(6.8, 26.2)	5.5	0.7	2.063*	(1.981, 2.172)	7.866	32.7	0.7	3.317*	(3.204, 3.364)
Pwani	7.8	11.9	7.6	4.3	(-5, 12.8)	2.8	3.1	-0.085	(-0.239, 0.014)	0.918	4.5	3.1	0.149*	(0.155, 0.545)
Rukwa	4.9	20.2	5.0	15.3*	(4.9, 25.4)	5.0	0.4	2.472*	(2.344, 2.465)	11.848	28.4	0.4	3.602*	(3.528, 3.684)
Ruvuma	15.0	26.6	15.1	11.5*	(1.4, 22.6)	5.5	5.3	0.131	(-0.113, 0.176)	1.140	19.1	5.3	0.806*	(0.755, 1.098)
Shinyanga	8.9	14.2	9.1	5	(-5.4, 16.5)	4.4	3.8	0.036*	(0.024, 0.29)	1.037	7.2	3.8	0.45*	(0.44, 0.802)
Simiyu	1.4	6.6	1.4	5.2*	(0, 12.9)	2.0	0.1	2.785*	(2.797, 2.924)	16.200	4.8	0.1	3.449*	(3.452, 3.727)
Singida	12.5	27.9	12.2	15.8*	(2.7, 27.8)	5.6	4.4	0.382*	(0.11, 0.391)	1.466	29.3	4.4	1.364*	(1.282, 1.602)
Tabora	11.0	17.1	11.1	6.1	(-3.8, 15.4)	3.2	3.2	-0.046	(-0.151, 0.131)	0.955	7.0	3.2	0.43*	(0.302, 0.455)
Tanga	21.8	26.6	21.7	4.9	(-7.3, 17.7)	4.8	7.8	-0.504*	(-0.615, -0.349)	0.604	7.1	7.8	-0.26*	(-0.711, -0.249)
National	9.8	17.7	9.8	7.9*	(5.8, 9.9)	0.2	0.2	-0.016	(-0.258, 0.028)	0.984	6.4	0.2	2.944*	(2.824, 3.023)

Note: * indicates p-value <0.05, MSE = mean squared error, CI = confidence interval

Supplementary Table 14: Coverage probability for each method for the indicator "Client had no problem with privacy from having others hear", by region

Region	Method 1: bootstrap	Method 2: svy1	Method 3: svy3
Arusha	0.87	0.834	0.834
Dar es Salaam	0.798	0.72	0.724
Dodoma	0.628	0.628	0.628
Geita	0.724	0.706	0.648
Iringa	0.852	0.824	0.824
Kagera	0.62	0.62	0.62
Katavi	0.646	0.646	0.646
Kigoma	0.666	0.666	0.666
Kilimanjaro	0.956	0.944	0.904
Lindi	0.808	0.768	0.768
Manyara	0.66	0.66	0.66
Mara	0.816	0.77	0.774
Mbeya	0.744	0.668	0.672
Morogoro	0.814	0.77	0.772
Mtwara	0.776	0.776	0.736
Mwanza	0.872	0.868	0.87
Njombe	0.628	0.628	0.628
Pwani	0.882	0.866	0.864
Rukwa	0.918	0.888	0.872
Ruvuma	0.772	0.74	0.744
Shinyanga	0.626	0.626	0.626
Simiyu	0.89	0.882	0.882
Singida	0.702	0.71	0.71
Tabora	0.818	0.768	0.768
Tanga	0.754	0.716	0.716
National	0.94	0.946	0.936

Legend

<0.91	0.91-0.93	0.93-0.95	0.95-0.97	>0.97	Not applicable

Supplementary Table 15: Coverage probability for each method for the indicator "Client had no problem with the cleanliness of the facility", by region

Region	Method 1: bootstrap	Method 2: svy1	Method 3: svy3
Arusha	0.966	0.966	0.95
Dar es Salaam	0.94	0.926	0.926
Dodoma	0.942	0.916	0.868
Geita	0.986	0.974	0.912
Iringa	0.934	0.92	0.896
Kagera	0.986	0.988	0.988
Katavi	0.968	0.918	0.84
Kigoma	0.93	0.9	0.888
Kilimanjaro	0.898	0.86	0.828
Lindi	0.93	0.92	0.88
Manyara	0.946	0.896	0.892
Mara	0.966	0.968	0.976
Mbeya	0.892	0.886	0.892
Morogoro	0.964	0.928	0.932
Mtwara	0.972	0.95	0.942
Mwanza	0.89	0.866	0.862
Njombe	0.958	0.934	0.92
Pwani	0.948	0.9	0.908
Rukwa	0.944	0.936	0.932
Ruvuma	0.934	0.894	0.888
Shinyanga	0.984	0.968	0.972
Simiyu	0.95	0.932	0.926
Singida	0.694	0.67	0.656
Tabora	0.974	0.972	0.964
Tanga	0.98	0.958	0.96
National	0.988	0.992	0.988

Legend

<0.91	0.91-0.93	0.93-0.95	0.95-0.97	>0.97	Not applicable

Supplementary Table 16: Coverage probability for each method for the indicator "Provider performed assessment of client weight during the ANC consultation", by region

Region	Method 1: bootstrap	Method 2: svy1	Method 3: svy3
Arusha	0.716	0.708	0.694
Dar es Salaam	0.934	0.864	0.81
Dodoma	0.896	0.88	0.88
Geita	0.796	0.772	0.706
Iringa	0.876	0.86	0.84
Kagera	0.862	0.848	0.828
Katavi	0.946	0.9	0.858
Kigoma	0.976	0.954	0.93
Kilimanjaro	0.756	0.722	0.72
Lindi	0.956	0.94	0.944
Manyara	0.974	0.93	0.9
Mara	0.89	0.874	0.83
Mbeya	0.96	0.856	0.854
Morogoro	0.922	0.894	0.89
Mtwara	0.876	0.876	0.762
Mwanza	0.852	0.842	0.842
Njombe	0.91	0.848	0.844
Pwani	1	0.884	0.888
Rukwa	0.882	0.86	0.848
Ruvuma	0.712	0.686	0.686
Shinyanga	0.86	0.794	0.786
Simiyu	0.954	0.938	0.932
Singida	0.69	0.686	0.674
Tabora	0.886	0.87	0.866
Tanga	0.936	0.9	0.898
National	0.974	0.98	0.972

Legend

<0.91	0.91-0.93	0.93-0.95	0.95-0.97	>0.97	Not applicable

Supplementary Table 17: Coverage probability for each method for the indicator "Provider performed RPR (syphilis test) laboratory investigation", by region

Region	Method 1: bootstrap	Method 2: svy1	Method 3: svy3
Arusha	0.868	0.816	0.782
Dar es Salaam	0.9	0.828	0.784
Dodoma	0.95	0.872	0.798
Geita	1	1	1
Iringa	0.932	0.86	0.81
Kagera	0.962	0.922	0.916
Katavi	0.96	0.926	0.856
Kigoma	0.926	0.862	0.806
Kilimanjaro	0.96	0.948	0.892
Lindi	0.974	0.962	0.932
Manyara	0.706	0.664	0.656
Mara	0.674	0.674	0.674
Mbeya	0.922	0.834	0.806
Morogoro	0.822	0.802	0.778
Mtwara	0.984	0.964	0.902
Mwanza	0.66	0.66	0.66
Njombe	0.976	0.936	0.904
Pwani	0.976	0.936	0.876
Rukwa	0.618	0.618	0.618
Ruvuma	0.882	0.864	0.848
Shinyanga	0.908	0.828	0.774
Simiyu	0.766	0.71	0.664
Singida	0.942	0.98	0.95
Tabora	0.802	0.722	0.716
Tanga	0.98	0.918	0.906
National	0.966	0.97	0.962

Legend

<0.91	0.91-0.93	0.93-0.95	0.95-0.97	>0.97	Not applicable

Supplementary Table 18: Coverage probability for each method for the indicator "Provider prescribed or gave tetanus toxoid injection", by region

Region	Method 1: bootstrap	Method 2: svy1	Method 3: svy3
Arusha	0.968	0.934	0.928
Dar es Salaam	0.986	0.824	0.814
Dodoma	0.982	0.986	0.984
Geita	0.98	0.978	0.962
Iringa	0.952	0.96	0.958
Kagera	0.978	0.936	0.93
Katavi	0.954	0.952	0.95
Kigoma	0.978	0.96	0.95
Kilimanjaro	0.966	0.95	0.926
Lindi	0.976	0.968	0.97
Manyara	0.962	0.958	0.97
Mara	0.98	0.97	0.968
Mbeya	0.928	0.89	0.888
Morogoro	0.924	0.928	0.93
Mtwara	0.982	0.972	0.976
Mwanza	0.93	0.886	0.882
Njombe	0.968	0.916	0.874
Pwani	0.976	0.906	0.902
Rukwa	0.976	0.938	0.942
Ruvuma	0.97	0.946	0.948
Shinyanga	0.972	0.942	0.956
Simiyu	0.964	0.968	0.958
Singida	0.978	0.99	0.984
Tabora	0.976	0.968	0.97
Tanga	0.972	0.94	0.928
National	0.988	0.994	0.994

Legend

<0.91	0.91-0.93	0.93-0.95	0.95-0.97	>0.97	Not applicable

Supplementary Table 19: Coverage probability for each method for the indicator "Facility has capacity to conduct hemoglobin testing on-site, by region

Region	Method 1: bootstrap	Method 2: svy1	Method 3: svy3
Arusha	0.986	0.908	
Dar es Salaam	0.776	0.718	
Dodoma	0.986	0.972	
Geita	0.932	0.88	
Iringa	0.978	0.904	
Kagera	0.96	0.84	
Katavi	0.974	0.95	
Kigoma	0.994	0.87	
Kilimanjaro	0.956	0.794	
Lindi	0.992	0.976	
Manyara	0.978	0.926	
Mara	0.98	0.87	
Mbeya	0.97	0.854	
Morogoro	0.978	0.95	
Mtwara	0.952	0.81	
Mwanza	0.968	0.89	
Njombe	0.988	0.888	
Pwani	0.984	0.956	
Rukwa	0.966	0.922	
Ruvuma	0.968	0.952	
Shinyanga	0.976	0.93	
Simiyu	0.96	0.93	
Singida	0.934	0.776	
Tabora	0.964	0.968	
Tanga	0.98	0.938	
National	0.982	0.982	

Legend

<0.91	0.91-0.93	0.93-0.95	0.95-0.97	>0.97	Not applicable

Supplementary Table 20: Coverage probability for each method for the indicator "Facility has TT vaccine in-stock", by region

Region	Method 1: bootstrap	Method 2: svy1	Method 3: svy3
Arusha	0.706	0.666	
Dar es Salaam	0.768	0.672	
Dodoma	0.862	0.86	
Geita	0.88	0.782	
Iringa	0.874	0.874	
Kagera	0.622	0.622	
Katavi	0.81	0.732	
Kigoma	0.806	0.74	
Kilimanjaro	0.788	0.716	
Lindi	0.96	0.956	
Manyara	0.696	0.632	
Mara	0.836	0.758	
Mbeya	0.948	0.94	
Morogoro	0.968	0.958	
Mtwara	0.924	0.89	
Mwanza	0.808	0.742	
Njombe	0.666	0.666	
Pwani	0.632	0.626	
Rukwa	0.914	0.884	
Ruvuma	0.948	0.928	
Shinyanga	0.618	0.618	
Simiyu	1	0.788	
Singida	1	0.736	
Tabora	0.972	0.97	
Tanga	0.954	0.94	
National	0.978	0.988	

Legend

<0.91	0.91-0.93	0.93-0.95	0.95-0.97	>0.97	Not applicable

Supplementary Table 21: Coverage probability for each method for the indicator "Facility has IPT drug in-stock", by region

Region	Method 1: bootstrap	Method 2: svy1	Method 3: svy3
Arusha	0.97	0.942	
Dar es Salaam	0.952	0.946	
Dodoma	0.978	0.968	
Geita	0.962	0.952	
Iringa	0.616	0.616	
Kagera	0.78	0.706	
Katavi	0.966	0.944	
Kigoma	0.976	0.964	
Kilimanjaro	0.938	0.914	
Lindi	0.97	0.95	
Manyara	0.976	0.952	
Mara	0.98	0.964	
Mbeya	0.96	0.948	
Morogoro	0.948	0.936	
Mtwara	0.974	0.96	
Mwanza	0.972	0.936	
Njombe	0.948	0.934	
Pwani	0.974	0.96	
Rukwa	0.976	0.966	
Ruvuma	0.966	0.956	
Shinyanga	0.968	0.946	
Simiyu	0.978	0.96	
Singida	0.982	0.966	
Tabora	0.97	0.96	
Tanga	0.99	0.978	
National	0.986	0.994	

Legend

<0.91	0.91-0.93	0.93-0.95	0.95-0.97	>0.97	Not applicable

Supplementary Table 22: Coverage probability for each method for the indicator "Facility has ITNs or ITN vouchers in-stock", by region

Region	Method 1: bootstrap	Method 2: svy1	Method 3: svy3
Arusha	0.972	0.926	
Dar es Salaam	0.974	0.906	
Dodoma	0.92	0.908	
Geita	0.888	0.888	
Iringa	0.976	0.93	
Kagera	0.938	0.888	
Katavi	0.874	0.874	
Kigoma	0.758	0.716	
Kilimanjaro	0.974	0.942	
Lindi	0.938	0.91	
Manyara	0.972	0.928	
Mara	0.91	0.89	
Mbeya	0.754	0.708	
Morogoro	0.936	0.92	
Mtwara	0.96	0.912	
Mwanza	0.972	0.824	
Njombe	0.978	0.938	
Pwani	0.812	0.756	
Rukwa	0.958	0.896	
Ruvuma	0.916	0.872	
Shinyanga	0.768	0.706	
Simiyu	0.872	0.872	
Singida	0.918	0.774	
Tabora	0.892	0.878	
Tanga	0.95	0.92	
National	0.986	0.984	

Legend

<0.91	0.91-0.93	0.93-0.95	0.95-0.97	>0.97	Not applicable

Supplementary Table 23: Average confidence interval (CI) length for each method for the indicator "Client had no problem with privacy from having others hear", by region

Region	Method 1: bootstrap	Method 2: svy1	Method 3: svy3	2*Log(bootstrap/svy1) *100	2*Log(bootstrap/svy3) *100
Arusha	0.0334	0.0370	0.0389	-21.0%	-28.4%
Dar es Salaam	0.0776	0.0972	0.0982	-27.0%	-30.3%
Dodoma	0.0134	0.0152	0.0153	-18.8%	-21.4%
Geita	0.2795	0.3598	0.3164	-40.0%	6.8%
Iringa	0.0589	0.0628	0.0537	-9.7%	13.8%
Kagera	0.0084	0.0091	0.0093	24.7%	18.0%
Katavi	0.0322	0.0419	0.0442	24.5%	-20.8%
Kigoma	0.0065	0.0070	0.0071	-7.4%	-11.3%
Kilimanjaro	0.2510	0.2593	0.2382	-8.1%	13.4%
Lindi	0.0886	0.1033	0.1043	-22.8%	-28.2%
Manyara	0.0112	0.0118	0.0117	-2.7%	-2.8%
Mara	0.0492	0.0528	0.0476	-7.0%	6.7%
Mbeya	0.1030	0.1058	0.1058	18.2%	12.2%
Morogoro	0.0791	0.0987	0.1004	-34.1%	-37.3%
Mtwara	0.1655	0.1888	0.0974	-20.3%	75.9%
Mwanza	0.0917	0.1121	0.1132	-33.4%	-35.6%
Njombe	0.1267	0.1680	0.1635	-31.6%	-30.3%
Pwani	0.0538	0.0572	0.0562	-11.1%	-6.2%
Rukwa	0.1515	0.1640	0.1505	-10.9%	10.6%
Ruvuma	0.0363	0.0395	0.0304	-13.7%	25.5%
Shinyanga	0.0981	0.1255	0.1208	-13.9%	-8.0%
Simiyu	0.0816	0.0945	0.0959	-23.5%	-25.7%
Singida	0.2668	0.3354	0.3195	-44.5%	-36.8%
Tabora	0.0435	0.0524	0.0531	-32.4%	-36.4%
Tanga	0.0916	0.1132	0.1130	-22.0%	-21.3%
National	0.0318	0.0349	0.0322	-18.4%	-1.7%

Legend

Largest CI length	-------->	Smallest CI length	Not applicable

Supplementary Table 24: Average confidence interval (CI) length for each method for the indicator "Client had no problem with the cleanliness of the facility", by region

Region	Method 1: bootstrap	Method 2: svy1	Method 3: svy3	2*Log(bootstrap/svy1) *100	2*Log(bootstrap/svy3) *100
Arusha	0.1915	0.2134	0.2050	-21.4%	-12.5%
Dar es Salaam	0.1672	0.1828	0.1835	-17.8%	-18.5%
Dodoma	0.1735	0.1955	0.1651	-19.6%	19.7%
Geita	0.2602	0.2665	0.2045	-4.2%	48.1%
Iringa	0.1358	0.1579	0.1496	-29.0%	-12.9%
Kagera	0.2929	0.3144	0.3051	-14.2%	-8.1%
Katavi	0.3624	0.3791	0.2698	-5.6%	64.5%
Kigoma	0.1320	0.1416	0.1334	-9.9%	4.0%
Kilimanjaro	0.2285	0.2307	0.2122	-1.2%	18.5%
Lindi	0.3383	0.3675	0.2448	-15.2%	61.0%
Manyara	0.0404	0.0331	0.0331	42.2%	38.0%
Mara	0.2371	0.2557	0.2468	-14.2%	-8.3%
Mbeya	0.3329	0.3712	0.3663	-15.6%	-14.2%
Morogoro	0.2218	0.2530	0.2507	-25.2%	-23.0%
Mtwara	0.1832	0.1688	0.1631	16.6%	22.7%
Mwanza	0.1540	0.1840	0.1811	-27.7%	-22.9%
Njombe	0.2240	0.2363	0.2291	-9.7%	-2.9%
Pwani	0.3068	0.3344	0.3269	-6.9%	-4.5%
Rukwa	0.2794	0.3097	0.2942	-19.3%	-9.1%
Ruvuma	0.0684	0.0729	0.0694	-6.3%	5.7%
Shinyanga	0.2424	0.2409	0.2334	1.7%	5.7%
Simiyu	0.2887	0.3294	0.3129	-24.0%	-12.8%
Singida	0.2701	0.3375	0.3188	-27.8%	-13.5%
Tabora	0.2241	0.2458	0.2367	-18.9%	-11.3%
Tanga	0.2122	0.2303	0.2295	-15.8%	-14.9%
National	0.0563	0.0606	0.0574	-15.6%	-4.6%

Legend

Largest CI length	-------->	Smallest CI length	Not applicable

Supplementary Table 25: Average confidence interval (CI) length for each method for the indicator "Provider performed assessment of client weight during the ANC consultation", by region

Region	Method 1: bootstrap	Method 2: svy1	Method 3: svy3	2*Log(bootstrap/svy1) *100	2*Log(bootstrap/svy3) *100
Arusha	0.4323	0.5957	0.5708	-56.6%	-46.4%
Dar es Salaam	0.0642	0.0454	0.0357	71.5%	119.9%
Dodoma	0.1787	0.2055	0.2058	-23.6%	-23.2%
Geita	0.4819	0.5800	0.4969	-32.6%	14.5%
Iringa	0.1245	0.1184	0.0845	11.3%	74.7%
Kagera	0.4694	0.5673	0.5430	-33.9%	-22.5%
Katavi	0.4760	0.5119	0.4079	-10.4%	32.7%
Kigoma	0.2858	0.3003	0.2780	-8.3%	8.7%
Kilimanjaro	0.3738	0.4174	0.3983	-4.9%	-5.5%
Lindi	0.4763	0.5182	0.4862	-15.4%	-4.6%
Manyara	0.2226	0.2313	0.2228	-3.9%	6.1%
Mara	0.3362	0.3715	0.2989	-16.9%	22.7%
Mbeya	0.7229	0.7472	0.7313	3.3%	7.3%
Morogoro	0.3096	0.3478	0.3399	-19.4%	-14.5%
Mtwara	0.3569	0.4245	0.3200	-29.4%	38.4%
Mwanza	0.5183	0.6539	0.6347	-33.9%	-20.4%
Njombe	0.2960	0.3466	0.3488	-20.5%	-22.4%
Pwani	0.0000	0.0000	0.0000	381.0%	380.0%
Rukwa	0.6026	0.7092	0.6651	-29.9%	-17.6%
Ruvuma	0.0994	0.1210	0.1208	-26.3%	-25.8%
Shinyanga	0.4677	0.5754	0.5406	-26.8%	-10.6%
Simiyu	0.5863	0.6585	0.6204	-22.7%	-10.8%
Singida	0.5031	0.6769	0.6346	-48.1%	-19.8%
Tabora	0.3443	0.3973	0.3859	-24.9%	-19.7%
Tanga	0.1809	0.1879	0.1813	-2.6%	6.0%
National	0.1297	0.1413	0.1344	-17.8%	-7.6%

Legend

Largest CI length	-------->	Smallest CI length	Not applicable

Supplementary Table 26: Average confidence interval (CI) length for each method for the indicator "Provider performed RPR (syphilis test) laboratory investigation", by region

Region	Method 1: bootstrap	Method 2: svy1	Method 3: svy3	2*Log(bootstrap/svy1) *100	2*Log(bootstrap/svy3) *100
Arusha	0.2819	0.3309	0.3002	-23.9%	2.9%
Dar es Salaam	0.0795	0.0565	0.0405	68.8%	132.2%
Dodoma	0.4027	0.3105	0.2404	58.3%	102.0%
Geita					
Iringa	0.2225	0.2161	0.1549	9.7%	73.8%
Kagera	0.2424	0.2680	0.2536	-18.4%	-6.1%
Katavi	0.3998	0.4162	0.2895	-4.2%	76.3%
Kigoma	0.2176	0.2198	0.1843	5.7%	55.4%
Kilimanjaro	0.5836	0.5935	0.4446	-2.7%	57.0%
Lindi	0.5188	0.5684	0.4857	-17.3%	16.0%
Manyara	0.4233	0.5396	0.5081	-24.3%	-4.9%
Mara	0.1302	0.1559	0.1544	-33.4%	-31.4%
Mbeya	0.0923	0.0676	0.0543	63.4%	113.7%
Morogoro	0.4839	0.5994	0.5622	-36.9%	-19.1%
Mtwara	0.5609	0.5842	0.4311	-6.0%	58.5%
Mwanza	0.0299	0.0273	0.0165	18.4%	119.6%
Njombe	0.6168	0.7178	0.6271	-25.4%	4.5%
Pwani	0.6236	0.7006	0.5663	-17.7%	29.2%
Rukwa	0.0312	0.0306	0.0303	6.3%	9.0%
Ruvuma	0.3926	0.4653	0.4414	-28.4%	-14.6%
Shinyanga	0.2407	0.2414	0.2175	13.1%	47.0%
Simiyu	0.3229	0.3826	0.3378	-19.8%	45.4%
Singida	0.5354	0.5577	0.4963	-5.5%	18.6%
Tabora	0.0824	0.0838	0.0654	-0.6%	50.9%
Tanga	0.4996	0.5470	0.4896	-11.5%	12.5%
National	0.0840	0.0885	0.0799	-11.2%	9.8%

Legend

Largest CI length	-------->	Smallest CI length	Not applicable

Supplementary Table 27: Average confidence interval (CI) length for each method for the indicator "Provider prescribed or gave tetanus toxoid injection", by region

Region	Method 1: bootstrap	Method 2: svy1	Method 3: svy3	2*Log(bootstrap/svy1) *100	2*Log(bootstrap/svy3) *100
Arusha	0.4193	0.4683	0.4501	-19.2%	-11.6%
Dar es Salaam	0.5381	0.5637	0.5463	20.1%	29.1%
Dodoma	0.3413	0.3716	0.3534	-16.0%	-6.1%
Geita	0.3996	0.4169	0.3631	-6.7%	20.2%
Iringa	0.5151	0.5912	0.5820	-24.1%	-22.3%
Kagera	0.4589	0.5136	0.4910	-19.8%	-10.5%
Katavi	0.3684	0.3868	0.3352	-9.8%	17.7%
Kigoma	0.3405	0.3623	0.3483	-11.5%	-3.4%
Kilimanjaro	0.5217	0.5365	0.4417	-3.3%	34.1%
Lindi	0.4653	0.5095	0.4891	-16.8%	-10.2%
Manyara	0.2998	0.3228	0.3380	-12.2%	-24.1%
Mara	0.4284	0.4388	0.4184	-3.4%	5.4%
Mbeya	0.6607	0.7265	0.7123	-15.8%	-12.4%
Morogoro	0.4802	0.5436	0.5320	-22.9%	-19.4%
Mtwara	0.4384	0.4571	0.4845	-7.6%	-21.2%
Mwanza	0.4948	0.5670	0.5543	-23.4%	-19.5%
Njombe	0.5061	0.4674	0.4025	19.7%	49.3%
Pwani	0.4992	0.5504	0.5258	-6.2%	3.9%
Rukwa	0.3848	0.4002	0.3966	-4.0%	-2.0%
Ruvuma	0.3911	0.4173	0.4138	-11.8%	-10.7%
Shinyanga	0.4609	0.5267	0.5116	-24.4%	-20.2%
Simiyu	0.5216	0.5552	0.5244	-10.4%	1.1%
Singida	0.3660	0.3864	0.3770	-5.6%	-4.0%
Tabora	0.4226	0.4229	0.4202	1.7%	1.9%
Tanga	0.5829	0.6919	0.6631	-30.5%	-21.8%
National	0.1246	0.1326	0.1279	-13.0%	-5.7%

Legend

Largest CI length	-------->	Smallest CI length	Not applicable

Supplementary Table 28: Average confidence interval (CI) length for each method for the indicator "Facility has capacity to conduct hemoglobin testing on-site", by region

Region	Method 1: bootstrap	Method 2: svy1	Method 3: svy3	2*Log(bootstrap/svy1) *100	2*Log(bootstrap/svy3) *100
Arusha	0.4908	0.5106		3.1%	
Dar es Salaam	0.2922	0.3485		-20.3%	
Dodoma	0.4783	0.5206		-17.4%	
Geita	0.4032	0.4459		-9.1%	
Iringa	0.3918	0.3809		18.0%	
Kagera	0.3529	0.3066		50.5%	
Katavi	0.3868	0.4105		-10.7%	
Kigoma	0.1702	0.0983		109.5%	
Kilimanjaro	0.3101	0.2880		36.2%	
Lindi	0.4563	0.4900		-12.9%	
Manyara	0.5564	0.6129		-13.4%	
Mara	0.4135	0.3579		44.2%	
Mbeya	0.2945	0.2769		29.9%	
Morogoro	0.4387	0.4480		0.5%	
Mtwara	0.2631	0.2393		45.0%	
Mwanza	0.4964	0.5210		3.6%	
Njombe	0.1926	0.1082		114.0%	
Pwani	0.5139	0.5361		-5.0%	
Rukwa	0.4282	0.4567		-10.6%	
Ruvuma	0.4426	0.4810		-14.5%	
Shinyanga	0.4997	0.5336		-8.5%	
Simiyu	0.4347	0.4785		-17.9%	
Singida	0.3213	0.3086		38.2%	
Tabora	0.4889	0.5289		-16.3%	
Tanga	0.4420	0.4627		-4.1%	
National	0.1019	0.1003		2.5%	

Legend

Largest CI length	-------->	Smallest CI length	Not applicable

Supplementary Table 29: Average confidence interval (CI) length for each method for the indicator "Facility has TT vaccine in-stock", by region

Region	Method 1: bootstrap	Method 2: svy1	Method 3: svy3	2*Log(bootstrap/svy1) *100	2*Log(bootstrap/svy3) *100
Arusha	0.2518	0.3156		-32.9%	
Dar es Salaam	0.2866	0.3367		-5.5%	
Dodoma	0.2358	0.2727		-25.0%	
Geita	0.2822	0.3239		-10.9%	
Iringa	0.2979	0.3594		-36.7%	
Kagera	0.0225	0.0223		0.8%	
Katavi	0.1560	0.1862		-33.0%	
Kigoma	0.1851	0.2072		-11.2%	
Kilimanjaro	0.2389	0.2809		-14.7%	
Lindi	0.4711	0.5191		-19.4%	
Manyara	0.2654	0.3198		21.1%	
Mara	0.2711	0.3126		-14.1%	
Mbeya	0.4447	0.4954		-21.4%	
Morogoro	0.5011	0.5478		-18.0%	
Mtwara	0.3405	0.3895		-27.2%	
Mwanza	0.0567	0.0529		12.2%	
Njombe	0.1817	0.2319		-46.1%	
Pwani	0.1832	0.2226		-25.3%	
Rukwa	0.3355	0.3798		-24.6%	
Ruvuma	0.4166	0.4612		-19.5%	
Shinyanga	0.2014	0.2569		-45.1%	
Simiyu	0.0000	0.0000		415.9%	
Singida	0.0000	0.0000		365.4%	
Tabora	0.4622	0.5001		-16.0%	
Tanga	0.4538	0.5080		-22.2%	
National	0.0848	0.0898		-12.2%	

Legend

Largest CI length	-------->	Smallest CI length	Not applicable

Supplementary Table 30: Average confidence interval (CI) length for each method for the indicator "Facility has IPT drug in-stock", by region

Region	Method 1: bootstrap	Method 2: svy1	Method 3: svy3	2*Log(bootstrap/svy1) *100	2*Log(bootstrap/svy3) *100
Arusha	0.5796	0.6562		-24.1%	
Dar es Salaam	0.5366	0.6290		-28.1%	
Dodoma	0.4212	0.4603		-16.8%	
Geita	0.5327	0.5970		-21.1%	
Iringa	0.0191	0.0193		-3.8%	
Kagera	0.2358	0.2726		-11.2%	
Katavi	0.3495	0.3759		-13.2%	
Kigoma	0.4942	0.5403		-18.1%	
Kilimanjaro	0.4361	0.4923		-21.0%	
Lindi	0.4749	0.5223		-18.3%	
Manyara	0.6004	0.6815		-24.3%	
Mara	0.5411	0.6088		-22.4%	
Mbeya	0.4911	0.5455		-21.0%	
Morogoro	0.4361	0.4863		-21.0%	
Mtwara	0.4877	0.5349		-18.8%	
Mwanza	0.5448	0.6196		-21.5%	
Njombe	0.4093	0.4590		-21.3%	
Pwani	0.5350	0.5895		-19.5%	
Rukwa	0.4798	0.5293		-19.8%	
Ruvuma	0.4925	0.5442		-19.7%	
Shinyanga	0.5694	0.6265		-18.6%	
Simiyu	0.4599	0.5091		-19.6%	
Singida	0.5843	0.6422		-18.4%	
Tabora	0.4573	0.4975		-16.9%	
Tanga	0.5243	0.5739		-18.2%	
National	0.1153	0.1217		-11.6%	

**Legend**	Largest CI length	-------->	Smallest CI length	Not applicable

Supplementary Table 31: Average confidence interval (CI) length for each method for the indicator "Facility has ITNs or ITN vouchers in-stock", by region

Region	Method 1: bootstrap	Method 2: svy1	Method 3: svy3	2*Log(bootstrap/svy1) *100	2*Log(bootstrap/svy3) *100
Arusha	0.1366	0.1078		45.6%	
Dar es Salaam	0.1077	0.0841		50.8%	
Dodoma	0.0505	0.0482		5.8%	
Geita	0.0726	0.0674		10.9%	
Iringa	0.1070	0.0853		43.7%	
Kagera	0.3850	0.4239		-9.8%	
Katavi	0.2104	0.2492		-34.6%	
Kigoma	0.1823	0.2106		-17.1%	
Kilimanjaro	0.4961	0.5510		-19.5%	
Lindi	0.0820	0.0687		32.7%	
Manyara	0.2023	0.1413		68.7%	
Mara	0.0875	0.0794		16.7%	
Mbeya	0.2025	0.2334		-10.2%	
Morogoro	0.3356	0.3765		-17.8%	
Mtwara	0.0926	0.0695		58.2%	
Mwanza	0.3954	0.3732		35.9%	
Njombe	0.1299	0.0945		60.9%	
Pwani	0.2199	0.2537		-16.7%	
Rukwa	0.1149	0.0867		53.9%	
Ruvuma	0.3052	0.3329		-7.7%	
Shinyanga	0.2487	0.2827		-2.8%	
Simiyu	0.0469	0.0460		1.4%	
Singida	0.3033	0.3236		12.2%	
Tabora	0.2542	0.2829		-15.1%	
Tanga	0.4079	0.4557		-19.0%	
National	0.0647	0.0682		-11.2%	

Legend

Largest CI length	-------->	Smallest CI length	Not applicable
